# Cannabis Use and Nicotine Vaping Cessation Outcomes: A Secondary Analysis of a Randomized Clinical Trial

**DOI:** 10.1001/jamanetworkopen.2025.47799

**Published:** 2025-12-12

**Authors:** Jodi M. Gilman, Corinne Cather, Harrison T. Reeder, Bryn Evohr, Gladys N. Pachas, Kevin M. Gray, Erin A. McClure, Randi M. Schuster, A. Eden Evins

**Affiliations:** 1 Center for Addiction Medicine, Department of Psychiatry, Massachusetts General Hospital, Boston; 2 Harvard Medical School, Boston, Massachusetts; 3 Biostatistics Center, Massachusetts General Hospital, Boston; 4 Medical University of South Carolina, Charleston

## Abstract

**Importance:**

Cannabis use is prevalent among adolescents and young adults who vape nicotine. It is not known if cannabis use affects nicotine vaping cessation success.

**Objective:**

To assess whether baseline frequency of cannabis use or cannabis use disorder (CUD) symptom severity was associated with nicotine vaping cessation in a randomized clinical trial.

**Design, Setting, and Participants:**

This secondary analysis of a randomized clinical trial with youth who vaped nicotine recruited at a single site in Massachusetts from June 2022 to May 2024. The trial included 3 groups receiving 12 weeks of varenicline treatment and placebo (both double-masked, paired with counseling), as well as single-masked referral to texting-app–based nicotine vaping cessation support (enhanced usual care [EUC]). Eligible participants were aged 16 to 25 years who reported vaping nicotine regularly and did not smoke tobacco.

**Exposure:**

Baseline cannabis use was assessed via self-reported number of days of cannabis use per week and with Cannabis Use Disorder Identification Test (CUDIT) scores.

**Main Outcomes and Measures:**

Biochemically verified 7-day point prevalence nicotine vaping abstinence at week 12. Logistic regression models estimated associations between baseline cannabis use and vaping abstinence. Interaction terms were evaluated to examine whether cannabis use moderated the effect of varenicline on nicotine abstinence.

**Results:**

Among the 261 participants randomized to nicotine vaping cessation treatment (mean [SD] age, 21.5 [2.0] years; 139 female [53%]), 28% (73 participants) reported no past-month cannabis use, 38% (100 participants) reported using cannabis more than 0 and less than 4 d/wk, and 30% (78 participants) reported using cannabis 4 to 7 d/wk. Cannabis use frequency was not significantly associated with nicotine vaping cessation (eg, 4 to 7 d/wk use vs no use: adjusted odds ratio [aOR], 1.14; 95% CI, 0.51-2.57; overall *P* = .20). Nor did cannabis frequency modify the effect of varenicline (eg, abstinence varenicline vs placebo or EUC among those with 4 to 7 d/wk use: aOR, 8.47; 95% CI, 2.78-28.25; vs among those with no use: aOR, 5.60; 95% CI, 1.97-17.06; overall interaction *P* = .32). Findings were similar for CUD symptom severity.

**Conclusions and Relevance:**

Among adolescents and young adults attempting to reduce or stop nicotine vaping, baseline cannabis use was not associated with nicotine vaping abstinence. Varenicline proved helpful for nicotine vaping cessation regardless of cannabis use, indicating that co-use of cannabis may not represent a barrier to successful nicotine vaping cessation treatment.

**Trial Registration:**

ClinicalTrials.gov Identifier: NCT05367492

## Key Points

**Question:**

Is cannabis use frequency or cannabis use disorder symptom severity associated with nicotine vaping cessation outcomes among adolescents and young adults?

**Findings:**

In this secondary analysis of a randomized clinical trial including 261 youth aged 16 to 25 years, baseline cannabis use frequency and cannabis use disorder symptom severity were not significantly associated with nicotine vaping abstinence at 12 weeks. The effectiveness of varenicline plus behavioral support for nicotine vaping cessation did not differ by cannabis use patterns.

**Meaning:**

These results suggest that cannabis use does not appear to hinder nicotine vaping cessation, suggesting varenicline remains effective for youth regardless of cannabis co-use.

## Teaser

This secondary analysis of a randomized clinical trial examines the association of cannabis use with nicotine vaping cessation among individuals aged 16 to 25 years.

## Introduction

Nicotine vaping is highly prevalent among adolescents and young adults and is now the most common form of nicotine use in this population.^1,2^ While some youth experiment with vaping transiently, a substantial subset develops patterns of regular use of vaped nicotine associated with nicotine withdrawal symptoms, failed quit attempts, and sustained nicotine addiction.^3^ Despite growing recognition of this public health concern, few evidence-based treatments exist for youth who wish to quit vaping nicotine.^4^ Even less is known about how co-occurring substance use may influence vaping cessation treatment outcomes in this priority population.

Cannabis use is common among youth who vape nicotine,^5,6,7,8,9,10,11,12^ with estimates that up to half of adolescents who vape nicotine also use cannabis,^13,14,15,16,17^ Preclinical evidence indicates a functional interaction between the endocannabinoid and cholinergic systems. Tetrahydrocannabinol (THC) is a partial agonist of high cannabinoid 1 (CB1) receptors, which are broadly distributed across reward-related brain regions.^18^ Activation of CB1 receptors by THC or synthetic agonists enhances nicotine’s rewarding properties, including increased nicotine-conditioned place preference^19^ and self-administration.^20^ Conversely, CB1 antagonists diminish these nicotine-related behaviors.^21^ These effects are bidirectional, as preclinical^19^ and clinical studies^22^ suggest that nicotine, in turn, enhances THC’s rewarding properties. Both THC and nicotine also engage mesolimbic dopaminergic pathways, which are central to reward processing and addiction.^20^ These overlapping neurobiological and behavioral pathways suggest that nicotine and THC may reciprocally enhance each other’s reinforcing and addictive properties.

Some studies report that co-use of cannabis and nicotine may lead to more persistent patterns of nicotine use and greater difficulty with tobacco abstinence,^23,24^ while others have found no association between cannabis use and nicotine cessation.^25,26^ Given these mixed findings, and the absence of any studies investigating specific relationships between nicotine vaping cessation outcomes and cannabis use patterns, there is a critical need to clarify the relationship between cannabis use and nicotine cessation outcomes to inform whether more effective, tailored interventions should be developed for individuals who regularly use both substances.

In a randomized, placebo-controlled trial of the efficacy of varenicline, a first-line pharmacotherapy for tobacco cessation,^27^ vs 2 forms of behavioral support for nicotine vaping cessation among 261 youth who did not regularly smoke tobacco (NCT05367492), varenicline was associated with significantly higher abstinence rates than either behavioral treatment.^4,28^ A majority of participants reported regular cannabis use at enrollment in the trial. Here we investigated whether baseline cannabis use frequency or cannabis use disorder (CUD) symptoms in the 30 days prior to enrollment were associated with nicotine vaping cessation outcomes. Given concerns about the potential cognitive and motivational effects of cannabis as well as the potential for cannabis co-use to increase the reward value of nicotine, we hypothesized that greater cannabis use frequency and severity would be associated with lower odds of vaping cessation, particularly among participants assigned to behavioral support alone.

## Methods

All participants provided informed consent (or assent with parental consent for minors), and the study protocol was approved by the Mass General Brigham institutional review board. This study followed the Consolidated Standards of Reporting Trials (CONSORT) reporting guideline.

### Study Design

**Figure 1.**
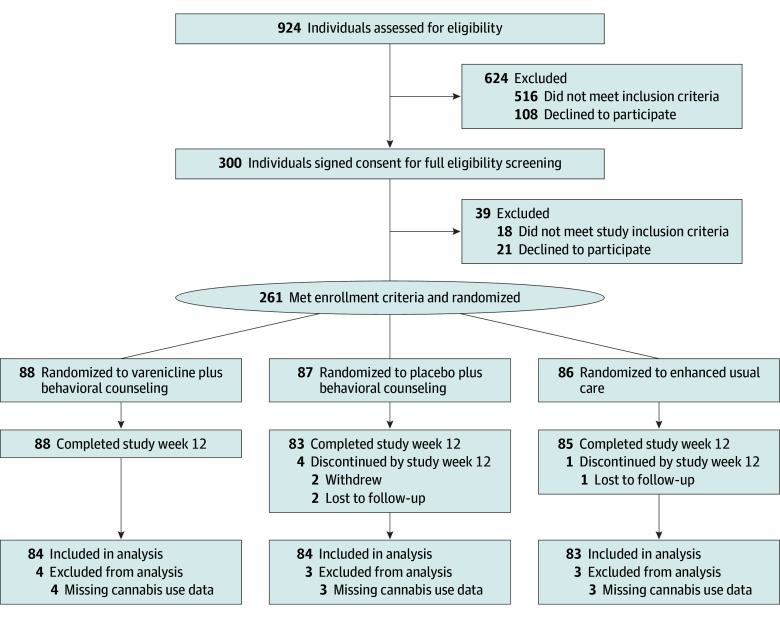
Participant Flow in the Study of Varenicline Added to Behavioral Treatment and Text Messaging Support for Vaping Cessation in Youth

This study is a secondary analysis of a randomized clinical trial that evaluated the efficacy of varenicline for nicotine vaping cessation among youth.^4^ The trial was registered on ClinicalTrials.gov (NCT05367492) (protocol and statistical analysis plan available in Supplement 1). Participants were randomized into 3 groups in a 1:1:1 ratio to receive 12 weeks of: (1) double-masked varenicline plus weekly, remotely delivered, individual behavioral counseling, (2) double-masked placebo plus behavioral counseling, or (3) single-masked referral to a text messaging nicotine vaping cessation support for youth^29^ (enhanced usual care [EUC]) (Figure 1).

### Participants and Setting

Participants were 261 adolescents and young adults aged 16 to 25 years who reported regular use of nicotine-containing e-cigarettes (defined as vaping 5 or more days per week in the past 90 days), exhibited nicotine dependence (defined as score of 4 or higher on the 10-item E-cigarette Dependence Inventory [ECDI]),^30^ and reported no prior regular tobacco smoking. Recruitment occurred through community advertisements, social media, and clinical referrals to a single site. At enrollment, participants self-reported age, sex, race, ethnicity, medical history, recent nicotine vaping, tobacco smoking, severity of nicotine dependence (assessed with the E-cigarette Dependence Inventory), and other drug use.^30^

### Cannabis and Alcohol Use Assessments

Baseline cannabis use was assessed in 2 ways. Cannabis use frequency was assessed using the Timeline Followback (TLFB) method,^31^ which captured self-reported cannabis use over the past 30 days. Cannabis use frequency was calculated as the average number of days of use per week and was categorized into 3 groups: zero days per week, up to 4 days per week, and 4 or more days per week. CUD symptom severity in the past 6 months was assessed using the 8-item Cannabis Use Disorder Identification Test–Revised (CUDIT-R),^32^ with higher scores indicating greater risk of CUD. CUDIT scores were categorized into clinical groupings corresponding to CUD risk: 0 to 7 indicating low risk, 8 to 11 indicating moderate risk, and 12 or higher indicating high risk and probable CUD. Finally, baseline alcohol use was assessed with frequency on the TLFB and alcohol use disorder severity was assessed using the Alcohol Use Disorder Identification Test (AUDIT).^33^

### Outcome Measures

The primary outcome of this analysis was point prevalence nicotine vaping abstinence at week 12, defined as self report of 7-day point prevalence nicotine vaping abstinence at the end of treatment (week 12) verified by salivary cotinine below 30 ng/mL using an oral fluid screening device (Abbott).^4^ Visits with self-reported abstinence but salivary cotinine concentration above 30 ng/mL were treated as nonabstinent. Visits with self-reported abstinence but missing bioverification were treated as abstinent, and visits missing both self-report and bioverification were treated as nonabstinent.

### Statistical Analysis

Descriptive statistics are reported by strata of baseline cannabis use frequency. Unadjusted counts and relative frequencies of abstinence are reported by baseline cannabis use frequency. Due to similar results between placebo plus behavioral counseling and EUC in the parent trial,^4^ we defined the control group in this analysis as those assigned to either the placebo or the EUC group.

We fit adjusted multivariable logistic regression models to assess associations between baseline cannabis use (use frequency and CUD symptom severity) and nicotine vaping abstinence at week 12. Adjusted models were fit, including age (as a continuous covariate), sex, baseline alcohol use severity (assessed with the AUDIT, modeled as a continuous covariate), and vaping cessation treatment group assignment. Separate models were run for categorical cannabis use frequency and for categorical CUD symptom severity (CUDIT) score.

For each model, an omnibus likelihood ratio test of the set of coefficients for categorical cannabis use is reported to test the hypothesis of no differences in odds of abstinence across cannabis use categories, adjusting for other covariates. For descriptive purposes, exploratory odds ratios (ORs) (relative to CUDIT score of 7 or below [ie, low risk for CUD], and zero d/wk of use, respectively) are reported with uncorrected 95% CIs. From each adjusted model we also report the adjusted odds ratio (aOR) estimate for AUDIT score, to investigate whether greater alcohol use would be linked to lower odds of nicotine vaping cessation. From each adjusted model we report the adjusted odds ratio estimate for varenicline (relative to placebo or EUC). Lastly, we fit a model adding interaction terms between treatment and cannabis use, from which we compute a likelihood ratio test of interaction to assess whether the effect of varenicline varies by baseline cannabis use. From each interaction model we also report exploratory aORs for varenicline vs placebo or EUC by baseline cannabis use level.

We performed several sensitivity analyses. First, we repeated all analyses excluding the varenicline group, with adjusted models omitting the corresponding treatment group variable. Second, we repeated these analyses modeling the association of abstinence with baseline CUDIT as a continuous, rather than a categorical, measure. Third, we repeated all analyses defining the outcome as continuous abstinence from study weeks 9 through 12, which was the primary outcome of the parent trial. Finally, as an exploratory analysis, in the subgroup who used cannabis at baseline, we compared cannabis abstinence rates over the past 30 days at week 12 across the 3 study groups (varenicline, placebo, and EUC) using a Fisher exact test.

Due to the low levels of missingness in the covariates, complete-case analyses were performed. All analyses were conducted using R software version 4.2.2 (R Project for Statistical Computing). The threshold for significance was *P* < .05 in 2-sided tests.

## Results

Among the 261 participants randomized to nicotine vaping cessation treatment (mean [SD] age, 21.5 [2.0] years; 139 female [53%]), 73 (28%) reported no past-month cannabis use, 100 (38%) indicated using cannabis more than zero and less than 4 days per week, and 78 (30%) reported using cannabis between 4 and 7 days per week (Table 1). The median (IQR) CUDIT score was 7 (3-13), with 131 participants (50%) of participants scoring 7 or below, 45 (17%) scoring between 8 and 11, and 83 (32%) scoring 12 or higher.

**Table 1.** Participant Characteristics by Cannabis Use Frequency in the 30 Days Prior to Enrollment

Characteristics	Overall, No. (%) (n = 261)	Cannabis use, No. (%)^a^		
		0 d/wk (n = 73)	>0 to <4 d/wk (n = 100)	≥4 d/wk, (n = 78)
Sex assigned at birth				
Male	122 (47)	29 (40)	46 (46)	42 (54)
Female	139 (53)	44 (60)	54 (54)	36 (46)
Assigned treatment group				
Varenicline plus behavioral counseling	88 (34)	26 (36)	36 (36)	22 (28)
Placebo plus behavioral counseling	87 (33)	23 (32)	30 (30)	31 (40)
Enhanced usual care	86 (33)	24 (33)	34 (34)	25 (32)
Age at baseline, mean (SD), y	21.5 (2)	21.9 (2)	21.4 (2.1)	21.2 (1.9)
Race				
Asian	46 (18)	18 (25)	19 (19)	7 (9)
Black	16 (6)	4 (5)	7 (7)	5 (6)
White	158 (61)	38 (52)	64 (64)	51 (65)
Multiple	30 (11)	9 (12)	8 (8)	10 (13)
Other^b^	11 (4)	4 (5)	2 (2)	5 (6)
Ethnicity				
Not Hispanic or Latino(a)	218 (84)	65 (89)	82 (82)	62 (79)
Hispanic or Latino(a)	43 (16)	8 (11)	18 (18)	16 (21)
Baseline e-cigarette Dependence Index, mean (SD)	13 (3.9)	13 (4)	12.6 (3.9)	13.8 (3.5)
Baseline CUDIT Score Category^c^				
0-7 (low risk)	131 (50)	68 (93)	50 (50)	11 (14)
8-11 (moderate risk)	45 (17)	2 (3)	20 (20)	20 (26)
≥12 (high risk/probable cannabis use disorder)	83 (32)	3 (4)	29 (29)	46 (59)
Missing	2 (1)	0	1 (1)	1 (1)
Baseline AUDIT Score, median (IQR)	6 (4-9)	5 (3-8)	7 (4-10)	6 (4-8)
Missing	4 (2)	0	2 (2)	2 (3)
Weekly frequency of alcohol use in 30 d prior to enrollment, median (IQR), d/wk	1.2 (0.5-1.9)	1.2 (0.5-1.9)	1.4 (0.5-2.2)	1.2 (0.5-1.9)
Missing	12 (5)	2 (3)	4 (4)	2 (3)

Abbreviations: AUDIT, Alcohol Use Disorder Identification Test; CUDIT, Cannabis Use Disorder Identification Test.

^a^ We note that 10 participants were missing cannabis use frequency at baseline. Complete case analyses of cannabis use frequency involved 247 participants (95%) of the full cohort.

^b^ Other race category includes 1 American Indian or Alaska Native participant and 2 Middle Eastern or North African participants, 7 Hispanic or Latino participants who listed Hispanic as both their race and ethnicity, and 1 participant who chose not to disclose race.

^c^ At baseline, 2 participants were missing CUDIT scores; complete case analyses of CUDIT scores included 257 participants (98%) from the full cohort.

**Figure 2.**
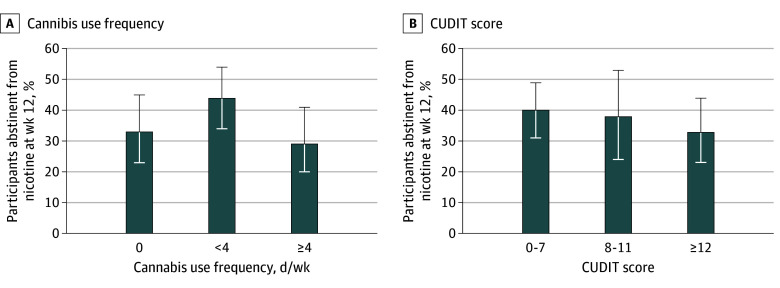
Nicotine Vaping Abstinence Rates by Baseline Cannabis Use Frequency and Cannabis Use Disorder Risk Overall abstinence rates reported without covariate adjustment. Error bars represent Wilson 95% CIs. CUDIT indicates Cannabis Use Disorder Identification Test. CUDIT scores were categorized into clinical groupings corresponding to CUD risk: 0 to 7 indicating low risk, 8 to 11 indicating moderate risk, and 12 or higher indicating high risk and probable CUD.

Nicotine abstinence rates based on cannabis use frequency were 33% (24 participants) for participants reporting no cannabis use, 44% (44 participants) for those using up to 4 days per week, and 29% (23 participants) for those using 4 to 7 days per week (Figure 2A), with no significant differences in conditional odds of abstinence among these groups (*P* = .20). Compared with participants reporting no cannabis use in the 30 days prior to enrollment, exploratory aOR estimates for abstinence were 1.87 (95% CI, 0.89-4.00) for those reporting using up to 4 days per week, and 1.14 (95% CI, 0.51-2.57) for those reporting use 4 to 7 days per week (Table 2). Findings were unchanged when excluding the varenicline group (eTable 1 in Supplement 2), and when defining the outcome as continuous abstinence over study weeks 9 to 12 (eTable 2 in Supplement 2).

**Table 2.** Association Between Cannabis Use, Alcohol Use, and Nicotine Vaping Abstinence

Measure	aOR (95% CI)	*P* value^a^
**Multivariable model: use days per week^b^**		
Cannabis use, d/wk		
0	1 [Reference]	.20
>0 and <4	1.87 (0.89-4.00)	
≥4	1.14 (0.51-2.57)	
AUDIT (5-unit increase)	0.91 (0.63-1.3)	.62
Varenicline (vs placebo or EUC)	10.03 (5.46-19.06)	<.001
**Multivariable model: categorical CUDIT scores^b^**		
CUDIT (categorical)		
0-7 (low risk)	1 [Reference]	>.99
8-11 (moderate risk)	1.02 (0.44-2.32)	
≥12 (high risk)	1.03 (0.52-2.06)	
AUDIT (5-unit increase)	0.93 (0.65-1.31)	.67
Varenicline (vs placebo or EUC)	9.74 (5.4-18.13)	<.001
**Multivariable model: continuous CUDIT^b^**		
CUDIT (5-unit increase)	1.03 (0.83-1.28)	.79
AUDIT (5-unit increase)	0.92 (0.65-1.3)	.65
Varenicline (vs placebo or EUC)	9.8 (5.43-18.23)	<.001

Abbreviations: aOR, adjusted odds ratio; AUDIT, Alcohol Use Disorder Identification Test; CUDIT, Cannabis Use Disorder Identification Test; EUC, enhanced usual care.

^a^ *P* values for categorical cannabis use variables from overall likelihood ratio tests, and *P* values for AUDIT, Varenicline, and continuous CUDIT variables from Wald tests.

^b^ Each multivariable model also adjusts for sex and age.

Nicotine abstinence rates were 40% (52 participants) for participants with CUDIT scores of 0-7, 38% (17 participants) for those with CUDIT scores of 8-11, and 33% (27 participants) for those with CUDIT scores 12 or higher (Figure 2B), with no significant differences in conditional odds of abstinence across these CUDIT score categories (*P* > .99). Relative to participants reporting a CUDIT score of 0 to 7, exploratory aOR estimates for abstinence were 1.02 (95% CI, 0.44-2.32) among those with CUDIT scores from 8 to 11, and 1.03 (95% CI, 0.52-2.06) among those with a CUDIT score of 12 or higher (Table 2). The findings were unchanged when excluding the varenicline group (eTable 1 in Supplement 2). Findings were also unchanged when analyzing CUDIT as a continuous score (5-unit increase: aOR, 1.03; 95% CI, 0.83-1.28; *P* = .79).

Although alcohol use was less frequent than cannabis use in this sample, we similarly examined the association between baseline alcohol use disorder (AUD) symptoms and nicotine vaping abstinence. Across all models, there was no significant association between AUD symptom severity as assessed with the AUDIT at baseline and vaping abstinence. For example, from the adjusted model for cannabis use frequency, a 5-point increase in AUDIT score was not associated with significant reduction in the odds for abstinence (aOR, 0.91; 95% CI, 0.63-1.30; *P* = .57) (Table 2).

For all models, there was an association between assignment to varenicline and higher conditional odds of abstinence than those assigned to placebo or EUC (Cannabis Use Frequency model: aOR, 10.03; 95% CI: 5.46-19.06; *P* < .001; CUDIT model: aOR, 9.74; 95% CI, 5.40-18.13; *P* < .001). No significant interaction effects were observed between treatment group and either cannabis use frequency (*P* for interaction = .28) or CUDIT score (*P* for interaction = .32), indicating that the magnitude of the beneficial effects of varenicline did not differ by cannabis use frequency or CUD severity (Table 3). In an abstinence model adjusting for CUDIT as a continuous measure, there was likewise a significant effect of treatment, varenicline vs placebo or EUC (aOR, 9.8; 95% CI, 5.43-18.23). There was also no significant interaction between varenicline and continuous CUDIT score (*P* = .48). These results were unchanged when defining the outcome as continuous abstinence over study weeks 9 through 12 (eTable 3 in Supplement 2). Among those with any baseline cannabis use, 6 of 45 participants (13%) in the varenicline group, 3 of 44 (7%) in the placebo group, and 6 of 48 (13%) in the EUC group reported 30-day abstinence from cannabis use at week 12 (*P* = .60), suggesting nicotine vaping treatment condition did not affect cannabis abstinence rates.

**Table 3.** Interaction Models of Varenicline by Baseline Cannabis Use

Multivariable model	Varenicline (vs placebo or EUC) group-specific, aOR (95% CI)	*P* value for interaction^a^
Use days per week^b^		
0	5.60 (1.97-17.06	.28
>0 to <4	18.71 (6.81-59.22)	
≥4	8.47 (2.78-28.25)	
Categorical CUDIT^b^		
0-7	8.36 (3.78-19.45)	.32
8-11	28.89 (6.29-180.72)	
≥12	7.18 (2.47-22.74)	

Abbreviations: aOR, adjusted odds ratio; CUDIT, Cannabis Use Disorder Identification Test; EUC, enhanced usual care.

^a^ Interaction *P* values for categorical cannabis use variables calculated from an overall likelihood ratio test of the interaction terms.

^b^ Each multivariable model also adjusts for sex, age, and continuous AUDIT score, and includes varenicline-cannabis interaction terms used to calculate group-specific adjusted ORs.

## Discussion

In this secondary analysis of a randomized clinical trial evaluating varenicline and behavioral treatment for nicotine vaping cessation in adolescents and young adults, we found that, contrary to our hypothesis, baseline cannabis use was not inversely associated with the likelihood of achieving nicotine vaping abstinence. Neither cannabis use frequency nor severity of potentially problematic cannabis use moderated nicotine treatment effects. The odds of nicotine vaping abstinence were similar across levels of cannabis use. In contrast, assignment to varenicline was associated with higher rates of nicotine vaping abstinence than behavioral vaping cessation support interventions, even after controlling for cannabis use. Assignment to varenicline did not affect rates of cannabis abstinence.

These findings are important because cannabis use is highly prevalent among youth who vape nicotine, and there are plausible mechanisms by which cannabis use could moderate nicotine cessation treatment efficacy. Cannabis use has been associated with greater substance use severity, psychiatric comorbidity,^34^ and, in some reports, generally diminished motivation^35^; therefore, we hypothesized that frequent or problematic cannabis use could interfere with efforts to quit nicotine vaping,^24^ especially in the context of behavioral treatment alone. However, these analyses do not support this hypothesis. The effectiveness of varenicline and of the behavioral vaping cessation support interventions appeared to be consistent across cannabis use frequency and severity categories, suggesting that ongoing cannabis use should not be viewed as a barrier to engaging youth in treatment for nicotine vaping cessation.

Our results also reinforce the efficacy of varenicline for vaping cessation in this population. In the parent trial, varenicline was associated with significantly higher rates of continuous abstinence than either placebo with behavioral counseling or app-based text messaging vaping cessation support.^4^ Here we show that the efficacy of varenicline for nicotine vaping cessation is consistent across cannabis use strata, as well as alcohol use strata, suggesting that varenicline may be an appropriate first-line treatment for youth who vape, regardless of co-occurring substance use.

Our findings did not demonstrate a significant difference in cannabis abstinence rates across treatment groups, suggesting that varenicline did not enhance cannabis cessation in this nicotine vaping cessation trial. Prior studies have explored potential benefits of varenicline for alcohol use disorder^36,37,38^ and for cannabis use disorder.^39,40^ These trials have reported mixed findings, with some suggesting varenicline was associated with reduced cannabis craving and quantity of cannabis used, but limited evidence for cannabis abstinence. Our results suggest that varenicline’s therapeutic effects for nicotine use disorder may not generalize to cannabis abstinence in individuals seeking treatment for nicotine vaping cessation who were not seeking treatment for cannabis use.^41^ Participants in this study did not enroll to attempt to quit using cannabis. Furthermore, no behavioral support for cannabis reduction or cessation was provided, which may be needed for varenicline to impact cannabis use outcomes.

### Limitations

This study has several limitations. Cannabis use and CUD symptoms were self-reported and may be subject to recall bias, although use frequency was assessed with validated TLFB methods and CUD symptoms with the validated CUDIT instrument. Although the sample was sufficiently powered to detect main effects, we may have been underpowered to detect more subtle interaction effects between varenicline treatment and cannabis use. However, there is no trend toward lower abstinence rates in those who used cannabis compared with those who did not. Low levels of alcohol use in the sample may have reduced our ability to detect an effect of alcohol on vaping cessation outcomes. We did not systematically assess the specific route of cannabis administration (eg, smoking, vaping, edibles), which may influence potential substitution effects, particularly among individuals who achieved nicotine vaping abstinence. Finally, the generalizability of our findings may be limited to youth who are motivated to try to reduce or quit nicotine vaping and do not smoke combusted tobacco regularly.

## Conclusions

Findings indicate that regular cannabis or alcohol use is not expected to diminish the effectiveness of offering varenicline for nicotine vaping cessation in youth. Given the urgent need to increase treatment availability for nicotine vaping cessation in adolescents and young adults,^42^ and widespread dual cannabis and nicotine use, these findings have implications for screening, treatment planning, and public health messaging. Future research should investigate whether integrated interventions targeting cannabis and nicotine co-use may yield additional benefit.

## Notes

Supplement 1. Trial Protocol and Statistical Analysis Plan

Supplement 2. eTable 1. Sensitivity Analysis Results for Association Between Cannabis Use and Abstinence Excluding Varenicline Group eTable 2. Sensitivity Analysis Results for Association Between Cannabis Use and Abstinence Using Continuous Week 9-12 Abstinence eTable 3. Sensitivity Analysis of Interaction Models of Varenicline by Baseline Cannabis Use, Using Continuous Week 9-12 Abstinence

Supplement 3. Data Sharing Statement
