# Supplementary material for: Cannabis Use and Nicotine Vaping Cessation Outcomes: A Secondary Analysis of a Randomized Clinical Trial
Source: JAMA Netw Open. 2025 Dec 12;8(12):e2547799. doi: 10.1001/jamanetworkopen.2025.47799 (PMC12701513; doi:10.1001/jamanetworkopen.2025.47799)
Supplement: Supplement 1. — Trial Protocol and Statistical Analysis Plan [file jamanetwopen-e2547799-s001.pdf]

Varenicline for Nicotine Vaping Cessation in Adolescents (ViVA) Statistical Analysis Plan

The ViVa Team

Version 2.2

First version: 01/12/2022

Latest version: 6/13/2024

NCT05367492

Glossary

Abbreviation	Label
BID	Two times a day
BMI	Body mass index
ECDI	E-cigarette Dependence Inventory
EUC	Enhanced usual care
FDA	U.S. Food and Drug Administration
MGH	Massachusetts General Hospital
MICE	Multiple imputation via chained equations
P+BC	Placebo combined with brief behavioral counseling
TIQ	"This is Quitting" vaping cessation program
V+BC	Varenicline combined with brief behavioral counseling

Study Design

We will evaluate the efficacy of a 12-week regime of varenicline, in addition to behavioral counseling and texting support, in increasing rates of vaping cessation among nicotine dependent adolescents, compared to (a) behavioral treatment and texting support and (b) enhanced usual care and monitoring.

The study will consist of a randomized three-arm, placebo-controlled, parallel-group, double-blind clinical trial:

1. [**V+BC arm**] Participants will receive the drug varenicline, in tablet form, up to 1 mg BID for 12 weeks. Additionally, participants will (a) attend QuitVaping behavioral support sessions

(completed in-person or via video-conferencing), once per week for 12 weeks, and (b) be encouraged to sign up for This Is Quitting (TIQ), a text message vaping cessation program for adolescents. We plan to assign up to 100 participants to this arm.

2. [**P+BC arm**]. Participants will receive placebo tablets, identical in appearance to varenicline, up to 1 mg BID for 12 weeks. Additionally, participants will (a) attend the QuitVaping behavioral support sessions once per week for 12 weeks, and (b) be encouraged to sign up for the TIQ program. We plan to assign up to 100 participants to this arm.
3. [**EUC arm**] Participants will be assigned to a single-blind monitoring only group and will receive no drugs nor attend behavioral support sessions. Participants will receive a brief encouragement to enroll in the TIQ program. We plan to assign up to 100 participants to this arm.

All participants will have weekly assessments to monitor vaping behavior, will receive advice to quit vaping at the first post-baseline weekly visit and be referred to the TIQ vaping cessation app. Other study-related outcomes will be assessed at the monthly visits (weeks 4, 8, 12, 16, 20, and 24).

Participants

We will recruit nicotine dependent adolescents who vape, do not smoke regularly, and are willing to try treatment to stop vaping. To do so, we plan to enroll up to 300 adolescents (and randomize as many as possible) in Greater Boston meeting these criteria. Our inclusion criteria are:

- Ages 16-25 (inclusive);
- Self-report of daily or near daily nicotine vaping for the prior ≥ 3 months, bio-verified by saliva cotinine >30 ng/ml.
- Nicotine dependence as defined by a score ≥ 4 on the 10-item E-cigarette Dependence Inventory (ECDI), and/or report persistent use despite negative consequences or prior failed quit attempts;
- Self-report of no regular combusted tobacco use in the past 2 months at enrollment and exhaled CO <10 ppm;
- Total body weight at enrollment ≥ 35 kg (77 lbs);

- Are competent and able to consent (if 18 or older), or have a parent or legal guardian who is able and willing to provide written informed consent (if under the age of 18);
- Able to understand study procedures and read and write in English;
- For participants who could become pregnant: negative urine pregnancy test at enrollment and agree to use effective contraception (e.g., abstinence, hormonal contraception, intra-uterine device, sterilization, or double barrier contraception) during the study.

Our exclusion criteria are:

- Use of a smoking cessation medication in the prior month (nicotine patch, gum, nasal spray, or inhaler, varenicline, bupropion);
- Unwilling to abstain during the study from using smoking cessation aids other than those provided by the study;
- Experienced a prior adverse drug reaction to varenicline;
- Report an unstable medical condition, epilepsy, or severe renal impairment;
- Had an inpatient psychiatric hospitalization and/or serious suicidal ideation or suicide attempt in the prior six months;
- Have active problem substance use (other than nicotine) severe enough to compromise ability to safely participate in the study protocol.
- Evidence of active problem substance use severe enough to compromise ability to safely participate, in the investigator's opinion;
- Unwilling to provide urine samples;
- Any condition or situation that would, in the investigator's opinion, make it unlikely that the participant could adhere safely to the study protocol;
- Ward of the state.

Randomization

Eligible participants will be randomly assigned in a 1:1:1 ratio, in blocks of 6, to V+BC (varenicline and behavioral counseling), P+BC (identical appearing placebo and behavioral counseling), or EUC (enhanced usual care). Randomization will be stratified by secondary or post-secondary school status. The randomization scheme will be computer generated by the Massachusetts General Hospital (MGH) Research Pharmacy by personnel with no other interactions with study staff or participants. Randomization codes will be held in the research pharmacy and made available to study investigators only in the case of urgent medical need. There will be separate randomization codes for apo-varenicline,

imported from Canada under a provisional FDA allowance, and domestic generic varenicline will full FDA approval. Treatment will be assigned according to study number, assigned sequentially to eligible participants who enroll in the trial and attend the baseline visit.

Masking/Blinding

Outcome assessors, data managers, and analysts will be blinded to treatment assignments. All study staff and participants will be blind to study medication assignment for the primary outcome comparing V+BC and P+BC. Interventionists and their clinical supervisors will be unblinded to assignment to behavioral counseling and will not be involved in assessments, data management, or data analysis.

Analytic Approach

Primary Outcome

The primary outcome for the study will be…

1. Cotinine-verified self-reported nicotine vaping abstinence from week 9 to week 12 of the study.
    a. [Time frame] Self-reported abstinence and urine or saliva samples will be collected during (a) the baseline visit, (b) every week from weeks 1 to 12 during the randomization interval, and (c) at weeks 16, 20, and 24 during the follow-up interval. The primary outcome will then consist of continuous abstinence status over weeks 9, 10, 11, and 12 for each participant converted to a binary value (1 = abstinent across all 4 study visits, 0 = non-abstinent for at least one of the 4 study visits).
    b. [Biochemical verification] Self-reported nicotine vaping abstinence will be considered verified given a semi-quantitative cotinine of less than 30ng/ml. For virtual sessions, participants will provide the sample during the videoconferencing session and will show results to the assessor. Those who report no vaping use since the last study visit but who have a cotinine of 30ng/ml or higher will be considered non-abstinent. Those who are missing cotinine values but self-report vaping will be considered non-abstinent.
    c. [Self-reported abstinence] Self-reported vaping will be assessed at all visits using the timeline follow-back (TLFB; Robinson, Sobell, Sobell, & Leo, 2014). The recall

period will be 60 days at enrollment and for all subsequent visits will be the interval between the prior and current visit.

d. [Randomization groups] The primary analysis will focus on V+BC and P+BC groups.

Covariates

We will use, at a minimum, 2 covariates in our analysis and missing data imputation:

- Biological sex at birth (male [referent] versus female).
- Summed scores from the ECDI (Foulds et al., 2015), a 10-item inventory with scores ranging from 0 – 20 (higher scores indicating greater E-cigarette dependence).

All covariates will be collected during the baseline visit.

Missing Data

Missing abstinence data for any of the study weeks 9, 10, 11, or 12 will be imputed 40 times using multiple imputation via chained equations (MICE; Azur, Stuart, Frangakis, & Leaf, 2011). Abstinence status will be imputed all non-missing time points and using the set of 2 covariates collected at the baseline visit described above. Imputation will be done via fully conditional specification with predictive mean matching using logistic regression. Following each imputation iteration, the 4 observed and/or imputed values per participant across the 4 study visits will be converted into the final binary outcome via the rules given above. For instances in which a participant only partially completed the baseline ECDI, missing responses to individual questions will be singularly imputed via an item response theory model fit to all non-missing responses: a binomial regression with population-level effects for items and a participant-varying intercept term.

Statistical Model and Tests

We will use a logistic regression model to assess continuous cotinine-verified self-reported abstinence across study weeks 9 – 12 for participants in the V+BC and P+BC arms (We will exclude participants in the EUC arm). Missing data will be imputed per the method specified above. We will then apply our logistic regression model to each set of observed and imputed data, and pool the regression estimates according to Rubin’s rule.

Our primary test of interest will be a dummy-coded contrast comparing the P+BC group (coded as 0) to the V+BC group (coded as 1). Statistical significance for the contrast between varenicline and

placebo will be determined using a z-test applied to the pooled mean estimate divided by the pooled standard error. We hypothesize that those assigned to V+BC will have significantly higher abstinence rates compared to those assigned to P+BC, which will be confirmed with $p < 0.05$ and an odds ratio greater than one.

The logistic regression model will include the 2 covariates assessed at baseline (biological sex and summed scores for the ECDI). Covariates will be standardized (mean-centered and scaled by the standard deviation). The analysis will be intent-to-treat; all subjects randomized at the beginning of the study will be included in the analysis, irrespective of whether they receive their assigned behavioral treatment or take their assigned medication.

Secondary Outcomes

The secondary outcomes for the study will be…

1. Cotinine-verified self-reported nicotine vaping abstinence from week 9 to week 24 of the study.
    a. [Time frame] Using self-reported abstinence (see primary outcome) and biochemical verification based on urine/saliva samples (see primary outcome) collected during weeks 9, 10, 11, 12, 16, 20, and 24, the secondary outcome will consist of continuous abstinence for each participant converted to a binary value (1 = abstinent across all 7 study visits, 0 = non-abstinent for at least one of the 7 study visits).
2. Cotinine-verified self-reported nicotine vaping abstinence at week 12 of the study.
    a. [Time frame] Using self-reported abstinence (see primary outcome) and biochemical verification based on urine/saliva samples (see primary outcome) collected during week 12, the secondary outcome will consist of 7-day point-prevalence abstinence for each participant converted to a binary value (1 = abstinent over 7 days ending at week 12, 0 = otherwise).
3. Summed scores on the Minnesota Nicotine Withdrawal Scale (MNWS; Cappelleri, Bushmakin, Baker, Merikle, Olufade, & Gilbert, 2005), a 9-item inventory with scores ranging from 0 to 36 (higher scores indicate greater severity of nicotine withdrawal).
    a. [Time frame] The inventory will be administered at baseline, every week from weeks 1 – 12, and weeks 16, 20, and 24.

4. Summed scores on the Questionnaire of Vaping Craving (QVC; Dowd, Motschman, & Tiffany, 2019), a 10-item inventory with scores ranging from 10 to 70 (higher scores indicate greater cravings to vape).
    a. [Time frame] The inventory will be administered at baseline, every week from weeks 1 – 12, and weeks 16, 20, and 24.
5. Summed scores on the General Distress subscale of the Mood and Anxiety Symptoms Questionnaire (MASQ-D30; Lin, Yung, Wigman, Killackey, Baksheev, & Wardenaar, 2014).
    a. [Time frame] The inventory will be administered at enrollment, baseline and monthly during weeks 4, 8, 12, 16, 20, and 24.
6. Number of participants who report one or more adverse events as measured by the Neuropsychiatric Adverse Events Interview (NAEI; Anthenelli et al., 2016).
    a. [Time frame] The interview will be conducted at enrollment, baseline, and monthly during weeks 4, 8, 12, 16, 20, and 24.

Missing Data

Missing outcome data for the 2 vaping abstinence outcomes and the 3 inventory measures will be multiply imputed.

Biochemically-Verified Vaping Abstinence

Missing abstinence data for any of the study weeks 9, 10, 11, 12, 16, 20, or 24 will be imputed 40 times using MICE via the same approach and covariates described for the primary outcome. Following each imputation iteration, for continuous abstinence over weeks 9 – 24, the 7 observed and/or imputed values per participant across the 7 study visits will be converted into the final binary outcome via the rules given above.

Inventory Measures

Missing summed scores for the MNWS, QVC, and the MASQ-D30 for any of the study weeks 1 – 24 will be imputed 40 times using MICE. Summed scores will be imputed using the set of 2 baseline covariates described above, as well as a participant's baseline summed score for the given inventory measure. Imputation will be done via fully conditional specification with predictive mean matching using linear regression.

Statistical Models and Tests

We will conduct 5 secondary analyses total for the 3 vaping abstinence outcomes, 3 secondary analyses and 3 exploratory analyses total for the 3 inventory measures, and a secondary analysis for adverse events.

Biochemically-Verified Vaping Abstinence

We will use a logistic regression model to assess (1) continuous vaping abstinence across study weeks 9 – 12, (2) continuous vaping abstinence across study weeks 9 – 24, and (3) 7-day point-prevalence cotinine-verified self-reported abstinence at week 12. Missing data will be imputed per the method specified above. We will then apply our logistic regression model to each set of observed and imputed data, and pool the regression estimates according to Rubin's rule.

The logistic regression model will include the 2 covariates assessed at baseline (biological sex and summed scores for the ECDI). Covariates will be standardized (mean-centered and scaled by the standard deviation). The analysis will be intent-to-treat; all subjects randomized at the beginning of the study will be included in the analysis, irrespective of whether they receive their assigned behavioral treatment or take their assigned medication.

We will conduct two types of analyses:

1. We will assess a single dummy-coded contrast comparing the P+BC group (coded as 0) to the V+BC group (coded as 1), excluding participants in the EUC group. Statistical significance will be determined using a z-test applied to the pooled mean estimate divided by the pooled standard error. We will conduct this analysis for our two secondary vaping abstinence outcomes.
2. We will assess a pair of dummy-coded contrasts comparing the EUC group (coded as 0) against (1) the V+BC group (coded as 1) and (2) the P+BC group (coded as 1). Statistical significance will be determined via an analysis of variance comparing a simpler null model (with contrasts for V+BC and P+BC fixed to zero) against the more complex model with both contrasts freely estimated. If the Wald test for the model comparison is significant, we will conduct post-hoc tests assessing the 3 possible comparisons between groups (EUC vs. P+BC, EUC vs. V+BC, and P+BC vs. V+BC). We will conduct this analysis for our primary vaping abstinence outcome as well as our two secondary vaping abstinence outcomes.

Table 1 summarizes all secondary analyses for the vaping abstinence outcomes. Tests will be deemed statistically significant for $p < 0.05$ following a correction using the Benjamini-Hochberg method across the 5 analyses.

Table 1: Secondary analyses for vaping abstinence outcomes

Vaping abstinence outcome	Contrast

Continuous abstinence	
(over weeks 9 – 12)	Omnibus test for EUC vs. V+BC, EUC vs. P+BC, and P+BC vs. V+BC
Continuous abstinence	Contrast of P+BC vs. V+BC (No EUC)
(over weeks 9 – 24)	Omnibus test for EUC vs. V+BC, EUC vs. P+BC, and P+BC vs. V+BC
Point-prevalence abstinence	Contrast of P+BC vs. V+BC (No EUC)
(week 12)	Omnibus test for EUC vs. V+BC, EUC vs. P+BC, and P+BC vs. V+BC

Inventory Measures

We will use a linear regression model to assess the post-baseline summed scores collected over weeks 1 – 12 (at weekly or monthly intervals) for (1) the MNWS, (2) the QVC, and (3) the MASQ-D30. The linear model will be estimated using generalized estimating equations (GEE; Liang & Zeger, 1986), providing estimates and standard errors robust to violations of distributional assumptions (e.g., normality) and heteroscedasticity.

The linear regression model will include covariates for (a) biological sex and baseline summed scores for the ECDI (see above), (b) a participant's baseline score on the given inventory measure, and (c) linear and quadratic time trend terms using number of days since the baseline visit. Covariates will be standardized (mean-centered and scaled by the standard deviation). The analysis will be intent-to-treat; all subjects randomized at the beginning of the study will be included in the analysis, irrespective of whether they receive their assigned behavioral treatment or take their assigned medication.

We will conduct two types of analyses:

3. We will assess a single dummy-coded contrast comparing the P+BC group (coded as 0) to the V+BC group (coded as 1), excluding participants in the EUC group. Statistical significance will be determined using a z-test applied to the pooled mean estimate divided by the pooled standard error. This will be a secondary analysis.
4. We will assess a pair of dummy-coded contrasts comparing the EUC group (coded as 0) against (1) the V+BC group (coded as 1) and (2) the P+BC group (coded as 1). Statistical significance will be determined via an analysis of variance comparing a simpler null model (with contrasts for V+BC and P+BC fixed to zero) against the more complex model with both contrasts freely estimated. If the Wald test for the model comparison is significant, we will conduct post-hoc tests assessing the 3 possible comparisons between groups (EUC vs. P+BC, EUC vs. V+BC, and P+BC vs. V+BC). This will be an exploratory analysis.

Table 2 summarizes all secondary and exploratory analyses for the inventory measures. Tests will be deemed statistically significant for $p < 0.05$ following a correction using the Benjamini-Hochberg method across the 3 secondary analyses and 3 exploratory analyses, respectively.

Table 2: Secondary and exploratory analyses for inventory measures

Inventory measure	Contrast	Exploratory
MNWS	Contrast of P+BC vs. V+BC (No EUC)	
	Omnibus test for EUC vs. V+BC, EUC vs. P+BC, and P+BC vs. V+BC	Yes
QVC	Contrast of P+BC vs. V+BC (No EUC)	
	Omnibus test for EUC vs. V+BC, EUC vs. P+BC, and P+BC vs. V+BC	Yes
MASQ-D30	Contrast of P+BC vs. V+BC (No EUC)	
	Omnibus test for EUC vs. V+BC, EUC vs. P+BC, and P+BC vs. V+BC	Yes

Adverse events

We will tally the number participants who report one or more adverse events during the NAEI interview across weeks 4, 8, and 12, looking specifically at the 2 x 2 contingency tables for (a) the P+BC group versus V+BC group, and (b) the EUC group versus the V+BC group. We will then assess whether the number of participants reporting one or more adverse events differs between the two groups using Fisher's exact test. Differences between groups will be deemed statistically significant for $p < 0.025$.

Sensitivity Analyses

Across our primary and secondary analyses, at a minimum we will conduct the following sensitivity analyses:

1. We will test whether our conclusion on efficacy is robust to the inclusion/exclusion of the two covariates (biological sex and nicotine dependence severity). We will rerun analyses excluding these covariates and see if conclusions change.

2. We will test whether our conclusion on efficacy is robust to the approach for imputation of missing data. We will (a) rerun the adjusted (i.e., with covariates) models on the observed data only and (b) rerun the adjusted models on a dataset with the observed data and all missing outcome data imputed as non-abstinent.
3. We will conduct a per protocol analysis by evaluating results among participants who use varenicline on most days in the trial (e.g., > 50 or 75%).

Power

Sample size was determined based on the assumption that use of varenicline for vaping cessation in adolescents would require an effect that at least doubled cessation rates over placebo to be worth the risks inherent in use of pharmacotherapy. Abstinence rates of 24% have been reported with TIQ alone in adolescents who vaped and were not necessarily dependent (Graham, Jacobs, & Amato, 2020; Graham, Amato, Cha, Jacobs, Bottcher, & Papandonatos, 2021). In this trial, requiring nicotine dependence to enroll, we postulate that addition of varenicline to behavioral counseling and TIQ will double abstinence rates. A sample size of 100 per arm, with 80 analyzable per arm, would yield power of 0.88 with a two-sided alpha of 0.05 with abstinence rates of 25% in the P+BC arm and 50% in the V+BC arm.

Software and Data Management

All analyses will be done using the statistical software R (version 4.1.1; R Core Team, 2021) and integrated development environment RStudio (version 2020.9.0.351; RStudio Team, 2021). Data will be prepared using the R packages ‘dplyr’ (version 1.0.7; Wickham, François, Henry, & Müller, 2021) and ‘tidyr’ (version 1.1.4; Wickham, 2021). Models will be fit using the R package ‘geepack’ (version 1.3-2; Højsgaard, Halekoh, & Yan, 2006). Missing data will be imputed using the R package ‘mice’ (version 3.13.0; Van Buuren & Groothuis-Oudshoorn, 2011). Reproducible code and de-identified data will be organized using the R package ‘targets’ (version 0.8.1; Landau, 2021).

References

Anthenelli, R. M., Benowitz, N. L., West, R., St Aubin, L., McRae, T., Lawrence, D., ... & Evins, A. E. (2016). Neuropsychiatric safety and efficacy of varenicline, bupropion, and nicotine patch in smokers with and without psychiatric disorders (EAGLES): A double-blind, randomised, placebo-controlled clinical trial. *The Lancet*, *387* (10037), 2507 - 2520. https://doi.org/10.1016/S0140-6736(16)30272-0.

Azur, M. J., Stuart, E. A., Frangakis, C., & Leaf, P. J. (2011). Multiple imputation by chained equations: What is it and how does it work? *International Journal of Methods in Psychiatric Research*, *20* (1), 40 - 49. https://doi.org/10.1002/mpr.329.

Buuren, S. van, & Groothuis-Oudshoorn, K. (2011). mice: Multivariate imputation by chained equations in R. *Journal of Statistical Software*, *45* (3), 1 - 67. https://doi.org/10.18637/jss.v045.i03.

Cappelleri, J. C., Bushmakin, A. G., Baker, C. L., Merikle, E., Olufade, A. O., & Gilbert, D. G. (2005). Revealing the multidimensional framework of the Minnesota nicotine withdrawal scale. *Current Medical Research and Opinion*, *21* (5), 749 - 760. https://doi.org/10.1185/030079905X43712.

Dowd, A. N., Motschman, C. A., & Tiffany, S. T. (2019). Development and validation of the questionnaire of vaping craving. *Nicotine and Tobacco Research*, *21* (1), 63 - 70. https://doi.org/10.1093/ntr/nty046.

U. S. Food and Drug Administration (2018). *Chantix (Varenicline tartrate)* (NDA 021928 / S-048). Retrieved from https://www.fda.gov/media/124220/download.

Foulds, J., Veldheer, S., Yingst, J., Hrabovsky, S., Wilson, S. J., Nichols, T. T., & Eissenberg, T. (2015). Development of a questionnaire for assessing dependence on electronic cigarettes among a large sample of ex-smoking E-cigarette users. *Nicotine & Tobacco Research*, *17*(2), 186 - 192. https://doi.org/10.1093/ntr/ntu204.

Graham, A. L., Jacobs, M. A., & Amato, M. S. (2020). Engagement and 3-month outcomes from a digital e-cigarette cessation program in a cohort of 27,000 teens and young adults. *Nicotine & Tobacco Research*, *22* (5), (859–860). https://doi.org/10.1093/ntr/ntz097.

Graham, A. L., Amato, M. S., Cha, S., Jacobs, M. A., Bottcher, M. M., & Papandonatos, G. D. (2021). Effectiveness of a vaping cessation text message program among young adult e-cigarette users: A randomized clinical trial. *JAMA Internal Medicine*, 181 (7), 923 - 930. https://doi.org/10.1001/jamainternmed.2021.1793.

Halekoh, U., Højsgaard, S., & Yan, J. (2006). The R package geepack for generalized estimating equations. *Journal of Statistical Software*, *15* (2), 1 - 11. https://doi.org/10.18637/jss.v015.i02.

Landau, W. M., (2021). The targets R package: A dynamic Make-like function-oriented pipeline toolkit for reproducibility and high-performance computing. *Journal of Open Source Software*, *6* (57), 2959. https://doi.org/10.21105/joss.02959.

Liang, K. Y., & Zeger, S. L. (1986). Longitudinal data analysis using generalized linear models. *Biometrika*, *73* (1), 13 - 22. https://doi.org/10.1093/biomet/73.1.13.

Benjamini, Y., & Hochberg, Y. (1995). Controlling the false discovery rate: a practical and powerful approach to multiple testing. *Journal of the Royal Statistical Society: Series B (Methodological)*, *57* (1), 289 - 300. https://doi.org/10.1111/j.2517-6161.1995.tb02031.x

Lin, A., Yung, A. R., Wigman, J. T., Killackey, E., Baksheev, G., & Wardenaar, K. J. (2014). Validation of a short adaptation of the Mood and Anxiety Symptoms Questionnaire (MASQ) in adolescents and young adults. *Psychiatry research*, *215* (3), 778 - 783. https://doi.org/10.1016/j.psychres.2013.12.018.

R Core Team. (2021). *R: A language and environment for statistical computing* (4.1.1). R Foundation for Statistical Computing. https://www.R-project.org.

Robinson, S. M., Sobell, L. C., Sobell, M. B., & Leo, G. I. (2014). Reliability of the Timeline Followback for cocaine, cannabis, and cigarette use. *Psychology of Addictive Behaviors*, *28* (1), 154 - 162. https://doi.org/10.1037/a0030992.

RStudio Team. (2021). RStudio: Integrated development environment for R (version 2020.9.0.351) [Computer software]. RStudio, PBC. http://www.rstudio.com.

Wickham, H., François, R., Henry, L. & Müller, K. (2021). dplyr: A grammar of data manipulation (version 1.0.7). [Computer software] Retrieved from https://cran.r-project.org/web/packages/dplyr/index.html.

Wickham, H. (2021). tidyr: Tidy messy data (version 1.1.4). [Computer software] Retrieved from https://CRAN.R-project.org/package=tidyr.

PARTNERS HUMAN RESEARCH COMMITTEE DETAILED PROTOCOL

VARENICLINE FOR VAPING CESSATION IN NON SMOKER VAPER ADOLESCENTS

Includes:

NCT04602494: Varenicline for Nicotine Vaping Cessation in Non Smoker Vaper Adolescents (Pilot)

NCT05367492: Varenicline for Nicotine Vaping Cessation in Adolescents (ViVA)

I. BACKGROUND

a. Background

The rapid rise in popularity of electronic nicotine delivery devices or ENDS (i.e., e-cigarettes) used to inhale vaporized nicotine solutions has effectively reversed 5 decades of denormalization of nicotine use among youth. While adolescent use of combusted tobacco products (cigarettes) has declined dramatically, vaping prevalence is on the rise and since 2014 has been the most popular form of nicotine use in youth. In 2018, 21% of U.S. high-school seniors reported vaping in the past month, a 10-point increase from 2017, which was the largest one-year increase in any substance used over the 44 years that Monitoring the Future has tracked adolescent substance use.[1] In 2019, over 25% of high-school and 11% of middle-school students nationally had vaped in the past 30 days. In many areas, over a third of high-school seniors use regularly. Most adolescents who vape have never smoked; an estimated 5.4 million middle- and high-school students in the U.S. recently used a vaping device, according to a federal survey released in December 2019, over 400% more than used cigarettes.[2] It is estimated that a million adolescents have become addicted to vaped nicotine.[3]

Health Consequences of Nicotine Vaping in Adolescent Non-Smokers: Empiric evidence supports that e-cigarette use should be discouraged among novice tobacco users.[4] Reduced nicotine exposure in adolescence is a benefit in itself, as nicotine exposure in adolescence induces lasting changes in brain function.[5–8] In a study in a Kaiser Permanente electronic medical record, among never-smokers, ENDS use was associated with greater odds of initiating smoking (OR = 7.41, 95% CI = 3.14-17.5).[9] There is also a growing consensus that ENDS increase risk of subsequent use of cannabis, alcohol, and other substances.[10] Therefore, cessation of ENDS use will reduce the risk for combusted tobacco use in this population[11,12] and probably reduce the risk for other problem drug use.[7,8,13,14] In addition, nearly a third of adolescents who vape have used an e-cigarette to vape cannabis, often high potency cannabis oils and concentrates.[15] Regular vaping has been associated with heavy metal and carcinogen exposure,[16] wheezing,[17] pulmonary inflammation,[18] impaired pulmonary gas exchange, and e-cigarette or vaping associated lung injury (EVALI).[19,20] A recent review concluded that it is not clear whether the respiratory health effects of ENDS are less than those of cigarettes.[21] ENDS cessation will reduce exposure of youth to these and other acute and long-term health risks.[22,23]

Desire to Quit: The prevalence of quitting smoking among youth is lower than adults.[24] The 2015 National Youth Risk Behavior Survey (YRBS) found that among 9–12 grade current cigarette smokers, 45.4% had tried to quit smoking during the prior year.[25] The National Youth Tobacco Survey found that, in 2015, the prevalence of having a past-year quit attempt among students in grades 9–12 was 57.8%, 4% higher than YRBS.[26] Among long-term vapers, 88% of whom were former smokers in a 2017 European survey, few intended to quit vaping.[27] In

contrast, responses to the 2019 National Youth Tobacco Survey administered by the Centers for Disease Control and Prevention suggest there is widespread youth interest in quitting e-cigarettes. Over half (54.5%) of middle- and high-school students who currently use e-cigarettes reported seriously thinking about quitting, and 52.9% reported trying to quit in the past year. Similarly, in a recent Truth Initiative survey, almost half of 15- to 24-year-olds who vape say quitting e-cigarettes is a 2020 resolution.[28]

Treatments Available but Not Yet Tested for Youth Vaping Cessation: Effective clinical interventions are clearly needed to help young non-smokers who vape to quit vaping. There are no controlled trials of vaping cessation interventions in the literature to date to our knowledge, though there are case reports of successful tapering with behavioral and pharmacologic support.[29,30] The Truth Initiative launched a free e-cigarette text message quit program, 'This is Quitting' (TIQ), for youth over age 13 years that has reportedly been used by 70,000 youth.[31] TIQ delivers tailored messages via text that give age-appropriate advice for quitters age 13 and older, including information about pharmacotherapy. Additionally, teen.smokefree.gov contains a section on vaping cessation.[32]

There are 7 FDA-approved medications for tobacco cessation: 5 nicotine replacement therapies (NRT), bupropion, and varenicline. All reduce nicotine withdrawal symptoms, which appear earlier and more frequently in youth than adults,[14] and include headaches, irritability, sleep difficulty, difficulty concentrating, and increased appetite.[33] Withdrawal symptoms, or fear of experiencing them, can be an important obstacle to quitting.[34] Cases of successful vaping cessation have been reported with varenicline,[29] and a preliminary case series in our group yielded more promising results with varenicline and behavioral support than NRT and behavioral support or behavioral support alone for adolescent vapers with nicotine dependence. Varenicline, a partial α-4 β-2 nicotinic receptor agonist that decreases nicotine withdrawal symptoms and craving, has been shown to be superior to placebo, NRT and bupropion for smoking cessation in multiple studies and populations,[35–37] and is well tolerated in adolescents over age 12.[38–40]

b. Prior clinical studies supporting the proposed research

Persistence of Adolescent Nicotine Vaping Despite Statewide Ban on All Vape Sales: In the fall of 2019, during a total ban on all vaping products in Massachusetts in response to the rapidly evolving EVALI public health crisis, we screened 7519 students in grades 6-12 in 11 schools in Greater Boston for tobacco, alcohol, and cannabis use and identified 422 students who reported daily nicotine vaping despite the ban. Amongst middle-school students surveyed, 14 of 2253 (0.6%) reported daily vaping, among those in grades 9-12, 408 of 5266 (7.7%) students surveyed reported daily vaping, and an additional 1228 (23.3%) reported vaping at least weekly in the prior 30 days. Thus, over 31% of high-school students in this sample of 11 schools in greater Boston reported frequent vaping, and 7.7% report daily vaping during a full ban on vaping in the state.[41]

High Desire to Quit but Low Success Rates with Conventional Behavioral Treatment: High school administrators at the 11 schools we surveyed in late 2019 reported vaping is a crisis in their schools and that disciplinary approaches are ineffective for reducing vaping in school. Those who have implemented behavioral interventions by school counselors report high uptake by students but low abstinence rates.

School Administrations Highly Motivated to Help Find Treatment to Mitigate Vaping Crisis in Schools: An email request was sent in December 2019 to school administrators statewide asking about interest in supporting vaping intervention studies we may propose. In the

next 2 days, just before winter recess, we received 170 letters of support from schools offering space and staff resources to assist with recruitment for and implementation of projects aiming to test and identify effective interventions for adolescent nicotine vaping cessation. Student health services in college settings similarly need guidance on vaping cessation treatment options.

Pharmacotherapeutic Treatment of Adolescents with Dependence to Vaped Nicotine, Open case series: A marked increase in treatment seeking amongst adolescents who vape nicotine, particularly among daily vapers with symptoms of nicotine dependence followed the statewide ban on all vape sales. We at the MGH Center for Addiction Medicine and colleagues at the Boston Children's Hospital Adolescent Substance Abuse Program (ASAP) have attempted to meet this increased demand with open label clinical treatment. To date, in an open case series, we have used a mobile telephone app for vaping cessation for adolescents who do not endorse nicotine withdrawal symptoms. For adolescent vapers with nicotine withdrawal symptoms, we have had success with varenicline and NRT combined with behavioral support. From Dec 2019-Jan 2020, we have treated 25 adolescents with varenicline and 24 with NRT. To date 18 of those treated with varenicline (72%) and 5 treated with NRT (21%) have stopped vaping.

Adolescent Non-Smokers Who Use ENDS have Significant Risk for Negative Outcomes: In 2018, we surveyed 1,628 students at two high schools in greater Boston on lifetime and current combusted tobacco, oral tobacco, and ENDS use, and past 3-month cannabis and alcohol use. Lifetime, past month, and daily ENDS use was endorsed by 29%, 21%, and 6% of students while 4%, 2% and 1%, respectively, endorsed combusted tobacco use. 70% of students who used tobacco, initiated use with vaped tobacco, and 9% of lifetime ENDS users reported subsequent regular cigarette use. Additionally, current frequent tobacco (ENDS or cigarette) use was strongly associated with frequent cannabis and alcohol use (ORs = 23.5 - 40.0; p's<0.0001), with no significant difference between ENDS and combusted tobacco users. This is consistent with prior reports that that onset of ENDS use before 9th grade increased the strength of the association between ENDS use and cigarette smoking, and cannabis, alcohol, and non-medical prescription drug use.[42] Overall, the health effects of prolonged nicotine vaping are unknown, which is why their use is discouraged among non-smokers,[42,43] particularly in adolescents whose risk for addiction is at a lifetime high.

Retention of School Recruited Adolescents in Abstinence Trials: In the past 2 years, we have enrolled 265 students from Greater Boston middle and high schools into a longitudinal investigation of the impact of a month of cannabis abstinence on neurocognition. Our retention rate of >85% demonstrates ability of our investigative team to recruit and retain a longitudinal cohort of adolescents with comparable research methods employed in a school-based setting, e.g., onsite drug testing, diagnostic interviews. Similarly, we enrolled 1169 smokers with schizophrenia in a community-based pragmatic smoking cessation trial, with retention rates of 80% at 3-year follow-up, further highlighting our team's strength in recruiting and retaining high risk cohorts in community settings.

c. Rationale and potential benefits to patients and/or society

There is no known benefit of ENDS in this population. Even among adult cigarette smokers from high-risk populations, there is debate and concern regarding the risk to benefit ratio of ENDS use. Moreover, youth who do smoke tobacco often engage in dual use of combusted cigarettes and ENDS.[44] Although e-cigarette vapor may be less hazardous than tobacco smoke, many recent reports challenge the idea that e-cigarette vapor is safe. Eighteen to 20% of EVALI patients report only using nicotine vapes prior to developing the idiopathic

vaping related serious pulmonary illness. Many volatile organic compounds identified in vaporized nicotine e-liquids are carcinogenic.[16] In addition, adolescent exposure to nicotine in animals has been shown to increase the addictive potential of other substances.[14] In our preliminary studies, adolescent vapers have elevated risk for use of alcohol and cannabis over those who do not use nicotine, not different from tobacco smokers. Although effective interventions are urgently needed to aid the estimated million adolescents in the US who have already developed nicotine addiction with daily ENDS use, to date, there are no published studies reporting effective treatments for nicotine vaping in adolescents.

II. SPECIFIC AIMS

We aim to test the hypothesis that varenicline added to behavioral and texting support will be well tolerated and improve vaping cessation rates over behavioral treatment and texting support and over enhanced usual care only in nicotine dependent adolescents who vape, do not smoke regularly, and are willing to try treatment to stop vaping. To do so, we propose to enroll up to 300 adolescents and randomize up to 240 in Greater Boston meeting these criteria into a 3-arm randomized, placebo-controlled, parallel-group study of varenicline up to 1 mg bid for 12 weeks added to behavioral and texting support specifically designed for teen vaping cessation compared with (1) behavioral and texting support and placebo and (2) enhanced usual care (EUC; i.e., monitoring and TIQ only).

Aim 1 (primary): To evaluate the efficacy of varenicline, added to behavioral and texting support, for cessation of vaped nicotine in adolescents with nicotine dependence.

Hypothesis 1.1: Those assigned to varenicline will have a higher rate of cotinine-verified, continuous nicotine vaping abstinence from study week 9 to end of treatment (weeks 9-12) (primary) and end of follow-up (weeks 9-24) (secondary) than those assigned to placebo, as operationalized by self-report of no nicotine vaping since the last study visit and salivary cotinine <30ng/ml at each study visit in the time frame designated.

Hypothesis 1.2 (secondary): Those assigned to varenicline will have a higher rate of cotinine-verified, seven-day point-prevalence nicotine vaping abstinence at end of treatment compared to placebo. Point prevalence abstinence is operationalized as self-report of no nicotine vaping since the last study visit and salivary cotinine <30ng/ml at study visit 12.

Hypothesis 1.3 (secondary): Those assigned to varenicline will have a higher rate of cotinine-verified, continuous nicotine vaping abstinence from study week 9 to end of treatment (weeks 9-12) and end of follow-up (weeks 9-24) than those assigned to EUC.

Hypothesis 1.4 (secondary): Those assigned to placebo will have a higher rate of cotinine-verified, continuous nicotine vaping abstinence from study week 9 to end of treatment (weeks 9-12) and end of follow-up (weeks 9-24) than those assigned to EUC.

Hypothesis 1.5 (secondary): Those assigned to varenicline will have a higher rate of cotinine-verified, seven-day point-prevalence nicotine vaping abstinence at end of treatment compared to EUC.

Hypothesis 1.6 (secondary): Those assigned to placebo will have a higher rate of cotinine-verified, seven-day point-prevalence nicotine vaping abstinence at end of treatment compared to EUC.

Hypothesis 1.7 (exploratory): Those assigned to varenicline will have an earlier onset of abstinence, longer latency to first lapse, longer latency to relapse, longer duration of abstinence, and greater total number of days of vaping abstinence than those assigned to placebo or EUC as

determined by using data ascertained daily with the emocha Mobile Health app (emocha) or REDCap surveys.

Aim 2 (secondary): To establish the safety and tolerability of varenicline in adolescents attempting to quit vaping nicotine.

Hypothesis 2.1: Nicotine withdrawal symptoms, craving, and negative mood symptoms will be less frequent and severe in the varenicline group than the placebo or EUCgroups.

Hypothesis 2.2: Those assigned to varenicline will have no greater incidence or severity of adverse events (AE) compared with those assigned to placebo or EUC during treatment and follow-up.

Aim 3 (exploratory): To assess if there are between group differences (based on intervention status and peer group vape use) in consumption of alcohol, combusted tobacco, cannabis, and non-medical prescription drugs during the treatment and follow-up periods.

Exploratory Hypothesis 3.1: Those assigned to varenicline will have less consumption of alcohol, combusted tobacco, cannabis, and non-medical prescription drugs and greater reduction in severity of nicotine dependence as assessed with the ECDI during the treatment and follow-up periods than those in the placebo or EUC groups.

Exploratory Hypothesis 3.2: Peer vape use and intervention status will interact. Participants enrolled in QuitVaping and involved in peer groups that endorse lower rates of vape use (as reported by their three closest friends) will endorse lower vape use at study conclusion.

Exploratory Hypothesis 3.3: Participants in the QuitVaping intervention will have more accurate perceptions of peers' vape use (as assessed by the correlation between participants' report and peers' report of peers' vape use).

PILOT STUDY 2020P002774-Pilot - NCT04602494

In 2021, during the national recall of varenicline in the US, the FDA provided temporary authorization for importation of apo-varenicline from the Canadian company, Apotex. A pilot vaping cessation trial was conducted using apo-varenicline or identical placebo in 4 study participants who met inclusion and exclusion criteria below except that the age range was limited to 18-25. In this pilot trial, all study procedures listed below were piloted, including the emocha platform for study medication adherence and daily report of vaping behavior and adverse effects and the novel, brief lay-counselor behavioral vaping cessation intervention.

Note: No IND was sought to enroll children into the trial with the apo-varenicline study medication, as it was being used under temporary FDA authorization. When US-made, generic varenicline was FDA approved (August 2021) and available for purchase through PAR Pharmaceuticals (November 2021), an IND was sought (December 2021) to enroll children ages 16 and 17 into the trial. This trial was deemed exempt in March 2022. When IND exemption was granted for enrolling children ages 16 and 17, IRB approval was sought for the main trial which will uses generic US made varenicline in participants ages 16-25.

III. SUBJECT SELECTION

a. Inclusion/exclusion criteria

Inclusion Criteria:

- Ages 16-25, inclusive;

- Self-report of daily or near daily nicotine vaping for the prior ≥ 3 months and semi-quantitative saliva screening for cotinine positive for recent nicotine use;
- Nicotine dependence as defined by a score ≥4 on the 10-item E-cigarette Dependence Inventory (ECDI), or report of persistent use despite negative consequences, or prior failed quit attempts;
- Self-report of no regular combusted tobacco use in the past 2 months at enrollment and exhaled CO <10 ppm;
- Total body weight at enrollment ≥35 kg (77 lbs);
- Report willingness to try varenicline to stop vaping;
- Able to understand study procedures and read and write in English;
- Have a parent or legal guardian who is able and willing to provide written informed consent (if under the age of 18);
- Competent and willing to provide written informed consent (if age 18+) or assent (if under 18);
- For participants who could become pregnant: negative urine pregnancy test at enrollment and agreement to use effective contraception (e.g., abstinence, hormonal contraception, intra-uterine device, sterilization, or double barrier contraception) during the study.

Exclusion Criteria:
- Use of a smoking cessation medication in the prior month (nicotine patch, gum, nasal spray, or inhaler, varenicline, bupropion);
- Unwilling to abstain during the study from using smoking cessation aids other than those provided by the study;
- Unstable medical condition, epilepsy, severe renal impairment;
- Inpatient psychiatric hospitalization in the prior 6 months, serious suicidal ideation or suicide attempt within 6 months of enrollment, recent active suicidal ideation or suicidal behavior identified at enrollment or baseline visits;
- Evidence of active problem substance use severe enough to compromise ability to safely participate, in the investigator's opinion;
- Prior adverse drug reaction to varenicline;
- Unwilling to provide urine samples;
- Any condition or situation that would, in the investigator's opinion, make it unlikely that the participant could adhere safely to the study protocol;
- Ward of the state.

Source of subjects and recruitment methods

Participants will be recruited via: 1) annual high school screenings conducted as part of the protocol, School Screening for Substance Use and Related Risk Factors PI: Schuster, Protocol #2021P001873, 2) direct physician referral from local MGH clinics and the MGB Healthcare network, 3) teacher, counselor, administrator, parent/guardian referral, 4) response to postings online, flyers, streaming services, through social media advertisements, and through traditional advertisements, 5) identification via research databases, such as the Research Patient Data Registry (RPDR) and Research Match. The MGB RPDR is a clinical data registry that can identify patients for clinical trials through EPIC records. We will run RPDR queries to find patients in the target age range with frequent vaping who are likely to be eligible for the current study. Research Match is a database of research volunteers developed by Vanderbilt University

and approved for use by the MGB IRB. Participants in high school will be identified through both school-based screening processes and non-school-based procedures. All potential participants, regardless of recruitment source, will complete a REDCap-based screener and/or telephone screen for eligibility. All participants will have the option to consent to receiving text messages from study staff. Text messages will be sent using Google Voice, and will be used to send deidentified study visit reminders to participants.

Ancillary Cohort

An ancillary cohort (N = up to 150) of participants' nominated top three peers will be recruited to participate in a single, remote visit after participants' study protocol is completed. Primary participants will be invited to provide the contact information (telephone number) of their top three peers at the week 24 visit (final study visit), as part of the Peer Network Structure measure. Participants will be informed that providing the contact information is voluntary, they are free to decline, and including contact information will not affect their participation in any way. Ancillary cohort recruitment will begin *after* the nominating participant completes the study.

Ancillary cohort participants will be recruited by blinded study staff via telephone (recruiting staff will only have access to the ancillary peer's name and phone number, they will not have access to information about the primary participant who provided their contact information or any data collected about that participant). Rates of ancillary participation will in no way influence the primary cohort's participation.

Inclusion criteria for the ancillary cohort:

- Able to understand study procedures and read and write in English;
- Have a parent or legal guardian who is able and willing to provide written informed consent (if under the age of 18);
- Competent and willing to provide written informed consent (if age 18+) or assent (if under 18).

Exclusion criteria for the ancillary cohort:

- Any condition or situation that would, in the investigator's opinion, make it unlikely that the participant could adhere safely to the study protocol;
- Ward of the state.

IV. SUBJECT ENROLLMENT

a. Methods of enrollment

School-Based Screening and Enrollment: Potential participants may be identified through an ongoing school screening process, School Screening for Substance Use and Related Risk Factors (PI: Schuster, Protocol #2021P001873), that involves school-wide assessments of all students in participating schools who were not opted out by their parents/guardians.

This assessment includes general demographic items and basic questions related to health behavior and psychological functioning. All students who are not withdrawn from the school-wide assessment by their parents/guardians and who wish to complete the questionnaire will also complete a separate form where they may provide their contact information and indicate their interest in being contacted for research studies. Questionnaires will be reviewed by MGH study staff, and all students who meet the criteria to participate in the study based on this initial

screening and who provided contact information will be contacted by MGH study staff to conduct a telephone screener or will be provided a link to complete the screener as a REDCap survey. The telephone screener will involve an explanation of study procedures as well as questions to screen for eligibility for the trial.

Students who express interest in participating in the study and are eligible based on the telephone screen will be scheduled for an in-person or virtual enrollment visit to sign consent (assent if under the age of 18) and complete eligibility screening procedures. A member of study staff will conduct informed consent procedures with at least one parent/guardian of participants under the age of 18 either in-person or via telephone/zoom prior to initiating study procedures. Parents/guardians will have the option to sign the ICF electronically if that is their preference.

Non-School-Based Screening and Enrollment: Participants identified through data registries like RPDR will receive a recruitment letter via Patient Gateway or by mail from study staff. The letter will not be sent to those who have opted out of receiving research invitations. MGB Research invitations will only be used to recruit participants aged 18 and older. Interested participants will reach out to study staff to complete a REDCap screener and/or telephone screener. Participants referred by self or other (e.g., in response to flyers, social media posting, clinic or school referral) will also complete a screener over the phone or via REDCap. The screener will involve an explanation of study procedures as well as questions to screen for eligibility for the trial. Potentially eligible participants interested in enrolling will be scheduled for an in-person or virtual enrollment visit to sign consent (assent if under the age of 18) and complete eligibility screening procedures. A member of study staff will conduct informed consent procedures with at least one parent/guardian of participants under the age of 18 either in-person or via telephone/zoom prior to initiating study procedures. Parents/guardians will have the option to sign the Informed Consent Form (ICF) electronically if that is their preference.

b. Procedures for obtaining informed consent

Waived Parental Consent for Phone Screening Only (for participants under age 18): We request a waiver of parental consent for those under age 18 for *phone screening only*, which is justified given the following:

The proposed phone screening involves no more than minimal risk to participants. During the phone screening, we will provide details about the study and ask potential participants if they have any questions or concerns about the study protocol. We will also ask participants basic demographic information as well as brief questions related to symptoms of psychopathology to assess eligibility. They will be told that all information will be kept strictly confidential and answers to screening questions will be stored separately from any identifying information. Participants are free to refuse to answer any questions. Participants will also have the option to complete the initial screening via a REDCap survey that will be accessible through the study's approved Massachusetts General Hospital Rally page. If participants are interested in the study and are eligible, we will collect parents/guardian's contact information so that we can obtain written parental consent for study enrollment. No study procedures will occur before written parental consent is obtained. We will also obtain written assent from the participant prior to the initiation of any study procedures.

The waiver of consent for phone screening will not adversely affect the rights and welfare of subjects. The waiver of parental consent for screening only will not adversely impact an adolescent's access to or the nature of any ongoing health services or primary care in any way nor adversely affect his/her rights or welfare. The study will be explained in detail to potential participants before any screening questions are asked, giving them a chance to decline screening if they are not interested in the study. They are also free to refuse to answer any screening questions that make them feel uncomfortable. They will be clearly told that written parental consent and adolescent assent will be required for enrollment in the active study phase and that answers to phone-based questions are for screening purposes only.

The research could not practicably be carried out with the waiver or alteration. This study could not practicably be carried out without a waiver of consent for phone screening to determine eligibility due to the burden that would be imposed on parents/guardians of potential participants that are not yet even known to be eligible and interested in study participation. This study utilizes a brief telephone screen to assess whether the potential participant is interested and eligible for the study. Specifically, obtaining written consent would significantly increase the time requirement for this brief screener. In our experience, the consent process generally takes approximately 30 minutes, while the brief screener should only take about 5-10 minutes for most participants. Requiring consent for screening would thus dramatically increase parent burden, and in many cases, this imposed burden would be unnecessary because it is not guaranteed that their child is interested or eligible for the study. Due to this increased parent/guardian burden, we expect that requiring consent for screening would greatly reduce enrollment and compromise the representativeness of the sample.

Participants and parents will be provided with additional pertinent information after participation. Prior to the first study visit, written parental consent will be obtained and parents/guardians will be provided with copies of the consent form which will fully detail the study protocol as well as any associated risks and benefits. They will be given contact information of key study staff. Similarly, study participants will be given a copy of the signed parental consent/study assent form.

The research involves no more than minimal risk to the privacy of the participants. This research involves no more than minimal risk to the privacy of participants. During the phone screening, only basic demographics and information to determine eligibility will be collected. The protocol fully details our plan to protect any identifying information from improper use and disclosure.

<u>**Written Parental Consent for Study Enrollment (<18 years of age):**</u> Parent/guardian contact information will be collected from potential participants who expressed interest in participating, who were found to be likely eligible via the initial screener (telephone or REDCap), and who are under the age of 18. After a participant under the age of 18 is determined to be likely eligible, study staff will reach out to the parent/guardian to obtain written informed consent. Parents/guardians can electronically sign the consent form and send the document back to study staff if they would prefer. For participants whose parent(s)/guardian only speak Spanish or Portuguese, written informed consent will be obtained using professionally translated Spanish or Portuguese consent forms. The opportunity to meet with study staff in person, virtually via video

conferencing, or over the phone will be encouraged and made available to all parents. Parents/guardians may reach out to MGH study staff with any questions or concerns prior to or after signing informed consent. Consent by parents or guardians will be documented. No participant under the age of 18 will be enrolled in the study until written parental informed consent is obtained.

<u>Written Participant Consent (18+ years of age) or Participant Assent (<18 years of age) for Study Enrollment:</u> Participant consent/assent procedures will occur at the enrollment visit, in which there will be a discussion of study procedures, potential adverse events, confidentiality, and voluntary participation. All participants will have the opportunity to ask questions regarding the study to study staff and/or doctoral-level member of the study staff. Participants must demonstrate understanding of the nature of this study, its potential risks, discomforts, and benefits before signing consent. All participants will have the ability to sign informed consent/assent electronically. No other study procedures will occur until informed consent/assent documents are signed. All participants as well as parents/guardians for participants under the age of 18 will be provided with a copy of their signed consent/assent documents and the Partners Privacy Statement. Minors who turn 18 while enrolled in the study will be re-consented to continue participation.

Ancillary cohort members will follow the same participant consent (if 18+) and parental consent/participant assent (if under 18) protocols as primary participants. Ancillary cohort members will sign the Ancillary Cohort consent form.

c. Treatment assignment, and randomization

Eligible participants will be randomly assigned in a 1:1:1 ratio, in blocks of 6, to varenicline, identical appearing placebo prepared by the MGH research pharmacy, or EUC (i.e., TIQ and monitoring with assessments only). Study staff and participants will both be blind to drug for those randomized to the varenicline and placebo arms. Randomization will be stratified by secondary or post-secondary school status. The randomization scheme will be computer generated by the MGH Research Pharmacy by personnel with no other interactions with study staff or participants. There will be separate randomization codes for apo-varenicline, imported from Canada under a provisional FDA allowance, and domestic generic varenicline with full FDA approval. The randomization codes will be held in the MGH research pharmacy, and available to study PIs only in the case of urgent medical need. Assessments will be conducted by study staff who are blind to study intervention. Treatment will be assigned according to study number, assigned sequentially to eligible participants who enroll in the trial and attend the baseline visit.

Special considerations for individuals living in the same households: More than one household member or family member will be allowed to participate in the study. Since there is a possibility that participants from the same household, voluntarily or not, share study medication, participants from the same household will be yoke randomized, eg assigned to the same intervention. Yoked randomization will ensure members of the same household or family members receive the same intervention and will reduce the risk for contamination.

Ancillary cohort participants will not be randomized. Protocols will be identical for all ancillary cohort members.

V. STUDY PROCEDURES

Participants will be randomly assigned to receive a 12-week course of varenicline, an identical placebo, or EUC only. Participants assigned to receive varenicline or identical placebo will receive individual behavioral vaping cessation sessions (held virtually by videoconferencing or in-person), and a digital platform to report vaping behavior, study medication adherence, and tolerability. Participants in EUC will be provided with explicit advice to quit and non-directive support. Participants in all three arms will complete assessments, conducted by raters blinded to study arm assignment, and will include samples for cotinine assays and self-reports of vaping and other drug use. Participants in all three arms will receive an introduction to TIQ and receive weekly monitoring of TIQ use from study staff.

a. Study visits and parameters to be measured

The enrollment visit will be conducted in person and will include a detailed description of the study, informed consent/assent, assessments to confirm eligibility, assessments of vaping and other drug use behavior, and medical and psychiatric diagnostic and symptom assessments. Eligible, consented participants will then be scheduled for a baseline assessment visit where they will receive study medications (for those randomized to varenicline or placebo arms), weekly visits which will include assessments (all participants) as well as the behavioral vaping cessation sessions (for those randomized to varenicline or placebo arms), and monthly assessment visits. Following completion of the 12-week intervention period, participants will be scheduled for follow-up visits at weeks 16, 20, and 24. Study visits will be conducted at our MGB location, or in a private room at a participant's local library or current school. When conducting a study visit at a site external to MGH, study staff will obtain verbal permission from library/school administrators to conduct study visits at a given location. When obtaining permission to conduct a visit, no identifying or specific information regarding the participant or the study will be mentioned. All study visits can be conducted in person or virtually. We have piloted procedures and have a standard operating procedure for sending materials to conduct urine drug and pregnancy screening during virtual visits. Study staff will include instructions for participants to perform the tests and hold the assays up to their device's camera so study staff can record urine screening results. Height and weight will be recorded per participant's report for remote visits. Carbon monoxide will not be recorded for virtual visits, and we will rely on participant reported combusted tobacco use. Medication will be shipped via priority mail if a visit where medication is distributed is done virtually. Blood pressure and heart rate will not be collected if a visit is virtual, as these are not necessary measures in prescribing varenicline or part of our primary outcomes.

<u>Time and Events Table</u>

Assessments	Enrollment	BL (Wk0)	Wk 1-3	Wk 4	Wk 5-7	Wk 8	Wk 9-11	Wk 12	Wk 16	Wk 20	Wk 24
**Screening**											
Consent	X	-	-	-	-	-	-	-	-	-	-
Demographics, Vaping history	X	-	-	-	-	-	-	-	-	-	-
**Primary outcomes**											
Salivary cotinine***	X	X	X	X	X	X	X	X	X	X	X
Timeline followback (TLFB)	X	X	X*	X	X*	X	X*	X	X	X	X
Daily vaping exposure y/n via REDCap (for those not randomized to EUC)	-	X	X	X	X	X	X	X	-	-	-
**Secondary/exploratory outcomes**											
MNWS	X	X	X	X	X	X	X	X	-	-	-
QVC	X	X	X	X	X	X	X	X	-	-	-
MASQ-D30	X	X	-	X	-	X	-	X	X	X	X
NAEI	X	X	-	X	-	X	-	X	X	X	X
Adverse events	-	X	X	X	X	X	X	X	X	X	X
**Other biomarkers**											
Urine NNAL	-	X	-	-	-	-	-	X	-	-	X
THC-COOH	-	X	-	-	-	-	-	X	-	-	X
CO	X	-	-	-	-	-	-	-	-	-	-
Pregnancy test	X	X	-	X	-	X	-	X	-	-	-
Drug screen	X	X	-	X	-	X	-	X	X	X	X
Vital signs	X	X	-	X	-	X	-	X	-	-	-
Height and weight	X	-	-	-	-	-	-	-	-	-	-
Saliva (genetics)	X	-	-	-	-	-	-	-	-	-	-
**Descriptions, effect modifiers, covariates**											
Medical History	X	-	-	-	-	-	-	-	-	-	-
Concomitant medications	X	X	X	X	X	X	X	X	X	X	X
ECDI	X	-	-	-	-	-	-	X	-	-	X
AUDIT	X	-	-	-	-	-	-	X	-	-	X
CUDIT	X	-	-	-	-	-	-	X	-	-	X
BRIEF-SR	X	-	-	-	-	-	-	X	-	-	-
C-SSRS	X	-	-	-	-	-	-	-	-	-	-
WTAR	X	-	-	-	-	-	-	-	-	-	-
UPPS-P	X	-	-	-	-	-	-	-	-	-	-
CHRT	-	X	-	X	-	X	-	X	-X	-X	-X
Flavor preferences	X	-	-	-	-	-	-	-	-	-	-
Peer and family influence on vaping	X	-	-	-	-	-	-	-	-	-	-
PATH Instruments: Vaping and substance use history, modified WTQ, MTQ	X	-	-	-	-	-	-	-	-	-	-
YSS	-	-	-	-	-	-	-	X	-	-	-
TIQ	-	X	X	X	X	X	X	X	-	-	-
Treatment assignment	-	-	-	-	-	-	-	X	-	-	-

Peer Network Stability	-	X	-	-	-	-	-	X	-	-	X
**Additional study procedures**											
Randomization	-	X	-	-	-	-	-	-	-	-	-
Drug distribution	-	X	X**	X	-	X	-	-	-	-	-
Quit Vaping Intervention	-	-	X	X	X	X	X	X	-	-	-

*Modified TLFB of past 7-day vaping during weekly visits **Week 2 only *** For weeks 1 thru 24, only administered to verify abstinence.

Ancillary cohort recruitment will begin *after* the nominating participant completes the study. Ancillary cohort contact information will be collected at participant’s Wk 24 visit. Ancillary cohort members will participate one time in a remote, questionnaire-only visit. They will not receive any intervention (behavioral, medication, or placebo). The remote visit will follow existing study protocols to ensure participant and data safety and privacy.

b. Drugs to be used varenicline or identical placebo (for participants randomized to varenicline or placebo arms)

Varenicline is a selective nicotinic acetylcholine receptor partial antagonist that binds primarily to the $\alpha4\beta2$ receptor subtype, thereby reducing both nicotine withdrawal and the reinforcing effects of nicotine. Due to its mixed agonist-antagonist properties, varenicline is effective at relieving nicotine craving and withdrawal during abstinence while blocking the reinforcing effects of nicotine in those who lapse.[45] The approved regimen of varenicline in adult smokers is 1 mg bid for 12 weeks, starting with a 1-week titration period (12-week course at 2 mg daily recommended).[46] A study in adolescents aged 12-17 years old (inclusive) found that steady-state systemic exposure in adolescent patients of body weight >55 kg, as assessed by AUC (0-24), was comparable to the adult population on the same dose.[39] When 0.5 mg bid was given, steady-state daily exposure of varenicline was, on average, higher (by approximately 40%) in adolescent patients with bodyweight ≤ 55 kg compared to the adult population.[39]

The dosing schedule is as follows:

For participants 16-17 years old, ≤ 55 kg:
- 0.5 mg once daily for 7 days, then
- 0.5 mg twice daily for 11 weeks

For participants 16-17 years old, >55 kg:
- 0.5 mg once daily for 3 days,
- 0.5 mg twice daily for 4 days
- 1 mg twice daily for 11 weeks

For participants 18+ years old, regardless of weight:
- 0.5 mg once daily for 3 days,
- 0.5 mg twice daily for 4 days
- 1 mg twice daily for 11 weeks

Medications will be adjusted to a lower dose if 1 mg bid is not well tolerated. Efficacy of doses lower than 1.0 mg per day have not been reported to our knowledge, thus participants who do not tolerate well the dose of varenicline or placebo 1.0 mg per day in single or divided doses will be discontinued from the study.

<u>Due to a shortage of varenicline in the US, the study medication in the **Pilot** study was provided by Apotex, Canada, apo-varenicline, which was on a temporary importation allowance from the FDA. US-made generic varenicline was FDA approved in August of 2021, made available for purchase in November 2021, and is currently used for the **main** study protocol.</u>

<u>Medication Distribution</u>: Study staff will distribute or send via priority mail varenicline or identically appearing placebo with instructions on how to take the study medication on the following schedule:

Baseline/Week 0: a 2-week supply
Week 2: a 2-week supply
Weeks 4 & 8: a 4-week supply

In the case of having participants who are traveling or experience unforeseen circumstances, we will coordinate with the research pharmacy to adapt the current distribution schedule to the participant’s needs to ensure they receive enough study medication.

<u>Medication adherence</u>: As part of the virtual weekly visit, participants will be asked to show their used and unused study medication each week to complete a weekly medication count to assess compliance. Missed doses will be discussed to try to ascertain the reason(s). Reasons for missed doses and/or patterns of missed doses will be incorporated into the vaping cessation sessions and every effort will be made to ensure proper subject dosing. Text messaging will encourage participants to take study medications as instructed. Participants will be instructed to bring all empty and unused study medication to in-person study visits. Unused medication will be cataloged by site staff to verify data reported by the subjects.

The emocha smartphone app will be used during the treatment period to remind participants via text message to take their study medication, document medication adherence and medication identification, and record daily assessments. Emocha will also be used for appointment reminders. When emocha is not available, participants will use REDCap to upload a time-stamped photo to confirm use of study meds. Recorded dosing will be incentivized with micropayments. Emocha and REDCap have embedded privacy protections.

Daily surveys are estimated to take 1-5 minutes each. To maximize compliance, we will adhere to best practice guidelines including training on the software at baseline and as needed throughout the study, ongoing compliance monitoring and follow-up with participants who demonstrate low adherence. Micropayments will be made to incentivize each survey completed ($1 per emocha video and survey completion, up to $2 daily).

c. <u>Devices to be used</u>: N/A

d. <u>Interventions</u> (for participant randomized to varenicline or placebo arms)

i. **QuitVaping** behavioral support sessions will be delivered weekly during the 12-week treatment phase via videoconference or in-person. QuitVaping is a manualized intervention based on the American Lung Association smoking cessation program[47] modified with teen friendly content and language for adolescent vaping cessation from the vaping section of teen.smokefree.gov[32] and the Truth Initiative, including:

- How to quit vaping: includes setting a quit date and standard quit date preparation modified for teen vaping;
- Your first day without vaping;
- Dealing with vape cravings, with standard behavioral methods for nicotine cravings and a prompt to call 1-800-QUIT-NOW or 1-877-44U-QUIT, or chat with an expert online using the National Cancer Institute’s LiveHelp service;
- Understanding your vaping triggers;
- Vaping addiction and nicotine withdrawal;
- Anxiety, stress, depression and vaping;
- How does vaping affect the environment? Your health?;
- Your likelihood of using other drugs or smoking;
- How to say no to vaping with friends;
- Information on the corporate exploitation of youth through the marketing of addictive products for profit, corporate support for vaping memes and corporate funding of social media influencers to promote vaping.

Before behavioral intervention sessions, participants will fill out assessments of vaping craving and collect saliva for cotinine testing (the latter only occurring if the participant self-reports nicotine abstinence since the last weekly visit). To refine and tailor these interventions to be maximally appealing to and effective for adolescents who vape, we will ask participants for weekly feedback on the appeal, relevance, and effectiveness of the educational topics and skills covered in the program. For participants randomized to the EUC arm, weekly sessions will only consist of non-directive support and assessments noted in the Time and Events Table, including saliva samples for cotinine (collected only to bio-verify self-reported nicotine abstinence).

CBT Structure

Time	Activities
5 mins	Check-in on adverse events, vaping exposure, and medication adherence
3 mins	New educational content
7 mins	Review of education material and skill/behavior goal

Prior to initiating the study intervention, interventionists will complete training conducted by Dr. Corinne Cather or other doctoral members of study staff on QuitVaping sessions. This will include didactic training on vaping and vaping cessation in adolescents, study methods, theoretical rationale, and background information on the behavioral and pharmacologic interventions. At the end of the training, leaders must achieve a minimum

score of 80% on the fidelity scale based on their facilitation of two simulated sessions before they lead a session.

e. Enhanced Usual Care (EUC)

i. **TIQ (This is Quitting)** is a free, publicly available text message vaping cessation program from Truth Initiative, designed specifically to help adolescents who vape nicotine quit. The program is grounded in theory-driven and empirically validated tobacco cessation treatment strategies for young people,[48] national cessation treatment guidelines,[49] the Mayo Clinic 5-E Model of Wellness Coaching,[50] and the Truth Initiative qualitative research and social media observations of young e-cigarette users.[51] TIQ is reported to have been used by over 70,000 youth in the first year after its launch in 2019.[52] TIQ texts messages with young people who have attempted to, or successfully, quit e-cigarettes, together with evidence-based tips and strategies to quit and stay quit. The language in TIQ is tailored based on the age and products used by the participant to give adolescents appropriate recommendations about quitting.

Participants who set a quit date receive messages for a week preceding it and 30 days afterward that include encouragement and support, skill- and self-efficacy building exercises, coping strategies, and information about the risks of vaping, benefits of quitting and cutting down to quit. Keywords “CRAVE,” “STRESS,” or “SLIP” provide on-demand support. Users can unsubscribe anytime by texting “STOP.” The teen version of the program (ages 13–17) refers to e-cigarettes as JUUL/JUULing, whereas the young adult version (YA, ages 18–24) uses several terms (e.g., vaping, e-cigarette).[52]

Participants in all three arms will be introduced to TIQ in the first session (week 0) and encouraged to sign up. Use of digital resources will be discussed and tracked in subsequent sessions along with troubleshooting of any problems. Any participant who does not have a compatible phone or for whom the texting program would pose a financial burden will be given a phone to use for the study.

f. <u>Data to be collected</u>

Questions derived from the PhenX Toolkit[53] will be collected during the phone screen to assess for demographics as well as other enrollment criteria, such as vaping and smoking history.

<u>Demographics</u> will be re-assessed by self-report during the enrollment visit.

<u>Medical History</u> will be assessed during the enrollment visit.

<u>Population Assessment of Tobacco and Health (PATH) Study Data Collection Instruments</u>[54]

The following questionnaires from PATH will be administered at the enrollment visit: Age of initiation, lifetime quantity and frequency of tobacco use, prior cessation attempts for vaped nicotine, other substance vaping, use of other forms of tobacco, cannabis products, alcohol, non-medical opioids, and other addictive substances.

Willingness to Quit (WTQ) vaping in the next 30 days will be queried at the phone screen and enrollment visit with the PATH questionnaire on serious thoughts about quitting all tobacco products.

Motivation to Quit (MTQ)[55] (PhenX ID=710501) is a single item measuring motivation to quit vaping among those who want to quit vaping. It will be administered at enrollment with the PATH questionnaire.

The Columbia-Suicide Severity Rating Scale Screener (C-SSRS)[56] will be used to assess suicidal ideation in the past six months.. This screener will be administered at enrollment and will help determine current suicidality incompatible with safe participation in the study.

Flavor Preference[57] Youth who vape are far more likely than adults to report using fruit, mint, and candy flavored vapes (OR=21(95% CI: 6, 75)).[58] This 5-item youth questionnaire will be used at enrollment to query e-cigarette flavor use and preference.

The Peer and Family Influence on Smoking[59] (PhenX ID=710901) will assess at enrollment smoked and vaped tobacco use by those in the participant's household, dormitory, suite or apartment, and perception of use by their immediate social networks. This 5-item measure can be self- or interviewer-administered. It is validated for ages 12-22, and its use up to age 26 has been recommended as has application of the questions to other tobacco use (e-cigarette and hookah).

The E-Cigarette Dependence (ECDI)[60] will be administered at enrollment, baseline, week 12, and week 24 to assess the severity of nicotine dependence, which has been reported to correlate significantly with cotinine as a biomarker of nicotine exposure. ECDI scores of ≥4, corresponding to at least mild nicotine dependence will be required for enrollment. The ECDI has been shown to be valid and reliable among e-cigarette users and dual users of e-cigarettes and combusted tobacco.

The Alcohol Use Disorders Identification Test (AUDIT)[61,62] was developed by the World Health Organization (WHO) as a simple screening tool to identify hazardous or harmful alcohol use. The AUDIT will be administered at the enrollment visit and at end of the treatment and follow-up phases of the study to detect change from baseline in harmful alcohol use.

The Cannabis Use Disorders Identification Test (CUDIT)[63] will be administered at the enrollment visit and at end of the treatment and follow-up phases of the study to detect change from baseline in symptoms of cannabis use disorder.

The Questionnaire of Vaping Craving (QVC)[64] is a brief, 10-item, valid and reliable measure of vaping craving that queries desire and intent to vape and anticipation of positive outcomes related to e-cigarette use that will be administered at enrollment, baseline, and weekly via REDCap.

Minnesota Nicotine Withdrawal Scale (MNWS)[65] is a valid, reliable, 9-item self-rated scale of urge to smoke (craving); depressed mood; irritability, frustration or anger; anxiety; difficulty concentrating; restlessness; increased appetite; difficulty going to sleep; difficulty staying asleep,

on an ordinal scale from 0 (not at all) to 4 (extreme). It will be administered at enrollment, baseline, and weekly via REDCap.

The Concise Health Risk Tracking Instrument (CHRT-SR)[66,67] will be administered to provide a sensitive assessment for change in suicidality to supplement open-ended adverse events queries at each study visit during the treatment phase. The CHRT-SR is a reliable and valid measure for examining severity of suicidal thoughts and associated risk factors and is sensitive to change in adolescents. It will be administered at baseline and each monthly assessment visit during the treatment and follow-up phases. Clinically trained study staff will follow-up on any positive responses.

The Mood and Anxiety Symptoms Questionnaire (MASQ-D30)[68] is a 30-item adaptation of the 96 item MASQ that is a quick, valid, and reliable assessment of depression and anxiety in adolescents that will be administered at enrollment, baseline, and each assessment visit.

Neuropsychiatric Adverse Events Interview (NAEI)[36] is a semi-structured interview designed to systematically assess the presence and severity of specific neuropsychiatric symptoms and will be administered at enrollment, baseline and at each assessment visits. This instrument will be asked in regards to the past six months (at enrollment) and since the last assessment visit (all other timepoints).

Adverse Events will be assessed weekly by the study staff via REDCap during the treatment phase and then monthly during assessment visits to assess the tolerability of the study procedures.

Concomitant Medications will be assessed by self-report at each visit.

Peer Network Stability: At baseline, V12, and 3-month follow-up, participants will be asked to list and rank up to 10 friends in order of closeness, using a modified version of the "concentric circles of closeness" sociometric measure used in. [80, 82] Participants will generate their friends' names by free recall to ensure (1) friends included in the study could come from any context and (2) participants designated only individuals whom they truly considered to be friends.[81] A friendship network instability index will be created by calculating the absolute value of the sum of each peer's rank change divided by the total number of peers the adolescent listed at the consecutive time points to account for differences in network sizes.[79] At the V24 visit (3-month follow-up) only, participants will be invited to provide telephone numbers for their top three nominated friends, to be recruited for the ancillary cohort.

Modified Mental Health Youth Satisfaction Survey(YSS) [69] is a 26-item self-rated survey of youth perception of services in 5 domains: access to services; appropriateness of services; cultural sensitivity; participation; and treatment outcome whose internal consistency ranges from 0.70 - 0.94. It has been widely used in state mental health systems to assess youth satisfaction with services and will be given at week 12 to assess participant satisfaction with the intervention.

The Impulsive Behavior Scale (UPPS-P) – Short[70] will be given at enrollment to assess five components of impulsivity, including sensation seeking, lack of premeditation, lack of

perseverance, negative urgency, and positive urgency. Scores on many of these factors have been shown to relate to risk behavior.

The Wechsler Test of Adult Reading (WTAR)[71] will be used to assess speech and dictation and derive an estimated IQ at enrollment.

The Behavior Rating Inventory of Executive Function—Self-Report (BRIEF-SR)[72] is a standardized 80-item self-report behavior rating scale that assesses impulsivity in cognition, behavior, and executive functioning skills. The BRIEF-SR will be administered at enrollment and week 12.

emocha Mobile Health is a mobile health platform that uses video technology and human engagement to monitor patient behavior and enable remote engagement in clinical trials. The emocha platform is globally compliant in terms of data protection standards, including GDPR ready, EU-US Privacy Shield certified, and HIPAA compliant. For those randomized to varenicline or placebo, emocha will be used to send twice daily reminders to take study medication and remotely monitor study medication dosing. Participants will record daily vaping either via emocha or REDCap.

Measures of Vaping abstinence: Saliva will be collected weekly during the treatment phase and then monthly for semi-quantitative cotinine measurement to verify nicotine abstinence in participants who self-report abstinence. Only at enrollment and baseline will cotinine be collected despite self-report to verify use. For in-person sessions, saliva will be collected before the session. For remote sessions, a handout with instructions, materials, and barcoded kits unique to participant will be mailed to participants to perform weekly before their weekly assessment visits. We developed SOPs for safe at home collection and mailing of biological specimens, a procedure we piloted during the closure of MGH clinical research facilities from March-June 2020. Semi-quantitative cotinine of <30ng/ml will validate self-reported nicotine vaping abstinence since the last visit. Those who report no vaping use since the last study visit but who have a cotinine >30ng/ml will be considered non-abstinent at that visit for the analyses.

Measures of smoking abstinence: Carbon monoxide (CO) in expired air will be measured at enrollment with a small, hand-held CO monitor. To do so, participants will blow into a straw attached to the device, a Bedfont Smokerlyzer II (Kent, England), following a 15-second breath-hold to verify self-report of non-smoking status. Expired CO <5 parts per million (ppm) is consistent with smoking abstinence. Assays for NNAL will be conducted on urine collected at baseline and weeks 12 and 24. NNAL <10pg/ml will be considered a more sensitive verification of self-report of no combusted tobacco use in the past 2-3 months. Participants with an NNAL of <10pg/ml at baseline and >10pg/ml during the study will be considered to have initiated combusted tobacco use during the trial.

Timeline Follow-Back (TLFB): The TLFB will assess tobacco vaping, smoking, cannabis, and alcohol use. It will be administered at enrollment, baseline, monthly, and follow-up visits. A modified TLFB of vaping in the past 7 days will be administered at weekly intervention visits.

Pregnancy test: Urine will be collected from biological females at enrollment, baseline, week 4, week 8, and week 12 for a pregnancy test.

Drug testing: Urine will be collected at enrollment, baseline, and monthly assessments to screen for recent use of cannabis, amphetamines, methamphetamine, cocaine, opiates, barbiturates, benzodiazepines, phencyclidine, methadone, and oxycodone. Quantitative assessment of THC metabolites in urine will be conducted at baseline, week 12, and week 24 to assess for new cannabis use or change in cannabis use during the trial.

Perceived treatment assignment: To assess participants' guess of their treatment assignment, participants will be asked, "what treatment do you think you received? This will be followed by one open-ended question: "Why do you think you received that treatment?" No clarifications will be asked, nor will participants be probed to expand on their answers. This question will be asked either at the end of the week 12 visit or their termination visit.

DNA Collection (saliva) and Analysis: DNA samples will be collected at enrollment using Oragene (OGR-500) saliva kits. Participants may be asked to provide a second sample if a recollect is recommended after DNA extraction (i.e., there is very little DNA in the sample). Participants are not required to provide another sample if they do not wish to do so. Once extracted, samples will be transferred to long term storage until genotyping. Samples will be stored with a unique participant ID.

GWAS Genotyping: The Broad Institute will perform genotyping (array-based) of subject samples and subsequent in-depth analysis of the data, which will allow us to detect alterations in the genome including point mutations, small insertions and deletions, chromosomal copy number alterations and translocations. These experiments are intended to help identify candidate genes involved in the physiopathology of neurological and psychological diseases. The molecular information generated from these samples will not be returned to subjects at any time.

Data from this study may result in communications in journals or at scientific meetings. Subjects will not be identified in those communications. To facilitate research, the genetic information generated may upon publication be deposited in protected databases (such as dbGAP) available only to bona fide researchers with specific scientific questions who promise to try not to identify individuals. The data will be sent to these banks in a coded manner and again will not contain any traditionally used identifiers such as name, address, phone number, or social security number. Although we cannot predict how genetic information will be used in the future, there are many safeguards in place, and we do not think that there will be further risks to patients' privacy and confidentiality by sharing such information with these banks.

The Broad Institute will not be involved in subject ascertainment. Prior to the transfer of biospecimen aliquots to the Broad Institute, samples will be re-encoded at the collaborator's institutions. No identifying patient information will be shared with Broad scientists at any time. Some limited clinical data will be obtained from collaborators and only de-identified clinical data will be shared with the Broad Institute.

Genetic Data Protection: In addition, steps will be taken to protect the confidentiality of genetic data as outlined:

1) All MGH study staff are trained to make confidentiality the first priority.
2) No genetic research data will be entered into the medical record.
3) The results of the genetic analyses will not be shared with participants, their family member(s), or unauthorized third parties.
4) Genetic data are encoded using coded identifiers. These codes, rather than personal identifiers, are used in any analytic datasets. The code key linking coded identifiers to personal identifiers is kept in an access-restricted, password-protected electronic file and is not shared with the genetic laboratories.
5) Consent forms are stored in locked cabinets apart from demographic and diagnostic data.
6) Samples and genetic data stored in the laboratory will be identified only by the code numbers and laboratory personnel will not have access to personal identifiers.
7) The most risk would be identification of individuals in the publicly shared database. To prevent this, computerized data files provided to other investigators will not include any of the HIPPA-defined personal identifiers. Published material will not identify subjects.

***Ancillary Cohort* Data to be Collected:**

Demographics will be assessed by self-report during the enrollment visit.

The Mood and Anxiety Symptoms Questionnaire (MASQ-D30)[68] is a 30-item adaptation of the 96 item MASQ that is a quick, valid, and reliable assessment of depression and anxiety in adolescents.

Timeline Follow-Back (TLFB): The TLFB will assess tobacco vaping, smoking, cannabis, and alcohol use. A modified TLFB of vaping in the month prior to associated primary participant's V24 will be administered.

COVID-19 Design Consideration

This protocol includes data collection and interventions that can be completed either in-person or virtually. These visits and assessments can be safely conducted in-person with ample precautions in place, and standard operating procedures to ensure the safety of the participants and staff have been written, reviewed, approved by our institution's COVID-19 safety officers, and are in place for our ongoing research protocols. Remote intervention and assessment visits may be required due to COVID-19 safety concerns, and we have piloted procedures for shipping study medication and specimen collection kits to participants' homes.

Early Termination and Relapse

Participants will be encouraged to attend all study visits, regardless of vaping status or ongoing use of study medication. Participants may voluntarily discontinue study procedures at any time for any reason. Participants may be discontinued if their continued participation in the

study presents an undue risk to their health as determined by the principal investigator or if they are unable or unwilling to follow study procedures. For any participant who discontinues treatment for any reason, an early termination visit will be conducted to assess vaping outcomes, nicotine craving and withdrawal, clinical symptoms, and adverse effects. Every effort will be made to contact participants who do not return for a scheduled visit. In any circumstance, every effort should be made to document subject outcome. Study staff will inquire about the reason for withdrawal, request the participant to return all unused investigational product(s), request the participant to return for a final visit, if applicable, and follow-up with the participant regarding any unresolved adverse events. Three telephone calls and one letter will be sent to participants who are lost to follow-up to schedule the early termination visit.

Participants will be terminated from the protocol if the research team determines that their continued participation is unsafe or not in their best interest (e.g., in circumstances where there is deterioration in their mental or physical health judged to require a change in medication or hospitalization). If a participant is discontinued, we will refer them to clinical resources for follow-up care or consultation, and we will ask them if this information that can be disclosed to their parents/guardians as applicable to help coordinate and facilitate care.

Study Compensation

Participants will be compensated $30 for the enrollment visit and all subsequent weekly and follow-up visits. Participants will be paid $50 (varenicline and placebo) or $100 (Enhanced Usual Care) for completion of monthly visits during the treatment period. Participants will be paid up to $2 per day (up to $168 total) for emocha medication adherence and REDCap survey completions (for those not randomized to EUC). All participants who complete the study will be automatically entered into a raffle for a study completion bonus. For a more detailed description of our payment schedule, refer to the table below. Participants will be reimbursed up to $15 for necessary travel related to in-person visits. Participants will be compensated for study visits either via MGB-issued check or using the MGB Advarra Participant Payment system, a cloud-based application that provides subjects with a reloadable Visa card that is credited with fixed stipends following specified data collection points. Participants will be informed of Advarra policy during the consent process. After agreeing to participate in the study, participants will also be asked to sign a “Advarra Participant Payment Card Acknowledgement of Receipt” form to certify their understanding of Advarra Participant Payment policies. The form will require participants to print and sign their name and provide their social security number. If the participant is under 18 years old, then parent/guardian information including date of birth, social security number, and address will be collected. Advarra acknowledgment forms will be stored in a locked study cabinet. Travel reimbursement as well as emocha/REDCap adherence will issued to participants as a lump sum via check at the conclusion of their study participation. Participants will be paid using gift cards if they cannot be paid via Advarra/check using a social security number/individual taxpayer identification number.

Ancillary participants will be compensated $30 for the one-time remote, questionnaire-based visit. All ancillary participants who consent will be compensated in full, regardless of how much of the visit they complete.

Payment Schedule

Visit/Week	Varenicline/Placebo	EUC
Enrollment	30	30
Weeks 0, 1, 2, 3	30	30
Week 4	50	100
Weeks 5, 6, 7	30	30
Week 8	50	100
Weeks 9, 10, 11	30	30
Week 12	50	100
Week 16, 20, 24	30	30
Emocha + surveys	168	0
**Total**	**738**	**720**
Raffle Bonus	450	468
**Grand Total**	**1188**	**1188**

Ancillary cohort: one-time $30 compensation for all participants after primary participants' Week 24 visit.

VI. BIOSTATISTICAL ANALYSIS

Statistical Design and Power:

Response estimates are based on 2 studies of the effect of varenicline or placebo plus brief weekly individual behavioral treatment for smoking cessation in adolescent smokers with nicotine dependence of a similar age range. One reported modest medication adherence (63% took 100% of dispensed doses) and high dropout rate (45%) which may underlie low abstinence rates (<10%) in both groups. We speculate that this trial under-estimates the therapeutic effect of varenicline for adolescent smoking cessation with more optimal treatment adherence. Additionally, potentially important participant characteristics, such as the proportion living with smokers, was not reported and may significantly impact abstinence outcomes in adolescents. In this trial, we will assess smoking and vaping in members of the household (or dorm) and in the primary peer group, and daily text message reminders to take medication and remotely observed dosing via emocha or REDCap will encourage medication adherence.

Continuous abstinence rates for weeks 9-12 of a 12-week intervention in the Pfizer report to the FDA in the varenicline arm ranged from 10-35%. Lowest abstinence rates (10%) were in older adolescents (ages 17-19) assigned to low dose varenicline (0.5 mg bid). In the proposed trial, adolescents who weigh >55kg, more likely amongst older adolescents, will be assigned to 1.0 mg bid as tolerated. Younger adolescents had numerically higher abstinence rates on 0.5 mg bid than 1.0 mg bid, regardless of weight, 16-21% versus 35%. This may have been due to tolerability issues. While there were no differences between any treatment arm in neuropsychiatric adverse events, the high dose group reported numerically more nausea, vomiting, and headache than the low dose and placebo groups. Those aged 17-19 with weight >55kg had better abstinence rates with 1.0 mg bid. Thus, we propose to dose by weight, not age, to employ flexible dosing as follows: All participants will be assessed for tolerability of 0.5 mg bid at the end of study week 1. Participants who weigh >55 kg who tolerate 0.5 bid with no complaints will be offered an increased dose of 1mg bid. Other participants will be continued at 0.5 mg bid.

The primary aim of this trial is to detect whether varenicline added to behavioral treatment significantly increases vaping abstinence rates compared with behavioral treatment alone. We have computed power over a range of outcomes. It is likely that varenicline will need to have a significant effect, such as doubling abstinence rates, to be considered appropriate for youth treatment. Such a significant increase in abstinence with varenicline over that achievable with low cost behavioral and digital intervention is clinically important because it would be warranted to justify the risk and expense of starting medications in this adolescent population.

Data Analysis:

Missing data:

Missing or incomplete outcome data from study week 9 to 12 will be imputed 40 times using multiple imputation via chained equations (MICE).[73] Outcome data will be imputed using three predictors collected at the baseline visit: biological sex (male versus female), environment (secondary school versus post-secondary school), and severity of nicotine dependence (the Penn State Electronic Cigarette Dependence Index, a summed score ranging from 0 – 20).[74] Imputation will be done via fully conditional specification with predictive mean matching using linear models for continuous data and logistic regression for binary variables.

Analyses of safety and efficacy will be conducted separately for participants assigned to Canadian manufactured, apo-varenicline or placebo (pilot) and for participants assigned to US generic Par Pharmaceutical manufactured varenicline (main trial). Analyses on the combined samples of Apo-varenicline and Par pharmaceuticals varenicline will be conducted if appropriate. If combined analyses are considered to be appropriate, these will be considered in the primary analyses.

Statistical method:

Hypotheses 1.1-1.6: We will use a logistic regression applied to the binary outcome for 4-week continuous abstinence at end of treatment (weeks 9-12; primary) and end of follow-up (weeks 9-24) (1 = abstinent, 0 = not abstinent). Participants whose status cannot be clearly determined due to missing data will have continuous abstinence status determined via imputation (see missing data plan). We will include three covariates measured as baseline: sex, environment (secondary school versus post-secondary school), and severity of nicotine dependence (the Penn State Electronic Cigarette Dependence Index, a summed score ranging from 0 – 20). Covariates will be standardized (mean-centered and scaled by the standard deviation). The analysis will be intent-to-treat; all subjects randomized at the beginning of the study will be included in the analysis, irrespective of whether they receive their assigned behavioral treatment or take their assigned medication. Missing data will be imputed per the method specified above. We will then apply our logistic regression model to each set of observed and imputed data, and pool the regression estimates according to Rubin's rule. Statistical significance for the contrast between varenicline and placebo will be determined using a z-test applied to the pooled mean estimate divided by the pooled standard error, where $p < 0.05$ will indicate that the addition of varenicline to behavioral and texting support is more effective than placebo for cessation of vaped nicotine in adolescent non-smokers with nicotine dependence.

The same analyses will be applied for secondary hypotheses comparing (1) varenicline to placebo for seven-day point-prevalence nicotine vaping abstinence at end of treatment (Hypothesis 1.2), (2) varenicline to EUC for 4-week continuous abstinence at end of treatment

and end of follow-up (Hypothesis 1.3), (3) placebo with behavioral treatment to EUC for 4-week continuous abstinence at end of treatment and end of follow-up (Hypothesis 1.4), (4) varenicline to EUC for seven-day point-prevalence nicotine vaping abstinence at end of treatment (Hypothesis 1.5), and placebo with behavioral treatment to EUC for seven-day point-prevalence nicotine vaping abstinence at end of treatment (Hypothesis 1.6).

Hypothesis 2.1: We will use a linear model applied separately to repeated, continuous weekly assessments of nicotine withdrawal symptoms, craving, and negative mood symptoms. The linear model will be estimated using generalized estimating equations (GEE), providing estimates and standard errors robust to violations of distributional assumptions (e.g., normality), and heteroscedasticity. The model will be applied to the summed scores at each time point per subject. The analysis will be intent-to-treat with missing data imputed per the method specified above. The key confirmatory effect will be a dummy-coded contrast comparing placebo (coded as 0) against varenicline (coded as 1). Exploratory analyses will also be conducted to evaluate varenicline versus EUC and placebo versus EUC. The model will include covariates for a) change over time (two terms encoding linear and quadratic trends), b) baseline levels of the given outcome, as well as c) sex, environment (secondary school versus post-secondary school), and severity of nicotine dependence. Covariates will be standardized (mean-centered and scaled by the standard deviation). P-values will be adjusted to avoid multiple comparison issues using the Benjamini-Hochberg method.

Hypothesis 2.2: We will conduct a Fisher's exact test differences by randomized group in the number (%) of participants who endorse an AE at least once in the 12-week treatment period. We will evaluate frequent AEs (e.g., that occur in at least 5% of the participants at any point between weeks 1 through 12 of the trial).

Sensitivity analyses: We will conduct 3 sensitivity analyses to examine the robustness of our conclusion for efficacy.

1. We will test whether our conclusion on efficacy is robust to the inclusion/exclusion of the 3 covariates (biological sex, environment [secondary school vs. post-secondary school], and nicotine dependence severity) – we therefore will rerun the analyses without the 3 covariates and examine whether the pooled estimate for the contrasts between varenicline, placebo, and/or EUC changes in sign and significance.
2. We will test whether our conclusion on efficacy is robust to the approach for imputation of missing data, examining whether the pooled estimate for the contrasts between varenicline, placebo, and/or EUC changes in sign and significance.
    a. We will rerun the adjusted (i.e., with covariates) models on the observed data only.
    b. We will rerun the adjusted models on a data set with the observed data and all missing outcome data imputed as non-abstinent.
3. We will test whether on conclusion on efficacy is robust to medication adherence by conducting a per protocol analysis. We will rerun the analyses only among participants who use varenicline on most days in the trial (e.g., 50-75%).

Software: All analyses will be conducted using the statistical software R (version 4.1.1).[75] Multiple imputation will be carried out using the R package 'mice' (version 3.13.0).[73] The GEE

method will be implemented via the R package ‘geepack’ (version 1.3-2).[76]

VII.. RISKS AND DISCOMFORTS

i. Varenicline Risks: Varenicline has been associated with significant adverse effects, including drowsiness and seizures, and must be used with caution while driving. Varenicline was previously thought to increase suicidality and neuropsychiatric side effects through similar mechanisms as bupropion and other antidepressants; however, the rate of neuropsychiatric adverse events has been shown to be no different between varenicline and placebo or bupropion or NRT patch in large-scale randomized controlled trial specifically examining these outcomes across an array of at-risk and general populations. Varenicline has not been approved for use in adolescents aged <18 years. However, recent studies have evaluated the pharmacology, safety, and tolerability of varenicline in adolescent smokers. The clinical pharmacology of varenicline in adolescent smokers was characterized in 1 single- and 2 multiple-dose pharmacokinetic (PK) studies in adolescent smokers. The most frequently reported adverse events in varenicline treated adolescent smokers were nausea, headache, vomiting, and dizziness. The incidence of nausea/vomiting increased with increasing varenicline exposure. Female adolescent smokers had a higher incidence of nausea relative to male adolescent smokers, regardless of dose, consistent with observations in adult smokers. Except for 1 case of severe nausea in a subject in the low-body-weight group (< 55 kg) on varenicline 0.5 mg once daily, all AEs were mild or moderate in intensity and resolved during the study. Psychiatric adverse events considered to be treatment-related were abnormal dreams (n = 2) and mild, transient anger (n = 1). No participants discontinued medication or had their dosage reduced due to an adverse event. A 12-week varenicline trial from a Pfizer-sponsored multisite study in 307 adolescent smokers (12-19 years of age) who were motivated to quit smoking reported no difference in tolerability between high-dose (1 mg bid) or low-dose (0.5 mg bid) varenicline treatment versus placebo.

To minimize risk of nausea, dosing will be recommended with 240 ml of water, and it is recommended that subjects eat prior to dosing. It is recommended that there be at least 8 hours between the morning and evening doses. Dosing may be reduced to 0.5 mg bid, temporarily discontinued, or stopped for subjects due to adverse events (e.g., nausea).

To minimize risk of rash, there have been rare post-marketing reports of serious rash occurring with use of varenicline. To minimize risk of allergic reaction, people with a history of hypersensitivity to varenicline will be excluded.

To minimize risk of neuropsychiatric adverse events, participants with a lifetime history of schizophrenia or bipolar disorder, an inpatient psychiatric hospitalization in the prior 6 months, a prior suicide attempt or serious suicidal ideation within 6 months of enrollment, or recent active suicidal ideation or suicidal behavior identified at enrollment or baseline visits will be excluded (see enrollment criteria). The study team is well prepared to identify and address urgent issues that may arise in the setting of new neuropsychiatric adverse events. Our investigators and group leaders are well-trained in psychotherapeutic interventions to help process and mitigate distress and have significant experience assessing and addressing patient safety. In addition, all study staff will be trained by a licensed psychiatrist (A. Eden Evins, MD, MPH) and licensed clinical psychologists (Corinne Cather PhD and Randi Schuster,

PhD) on identifying and responding to emotional distress in research participants. To ensure this process is robust, we have developed an extensive standard operating procedure (SOP) for safety monitoring to ensure clinical research coordinators are able to reliably recognize emergent suicidal ideation based on a clearly defined pattern of responses on self-report inventories. The most updated SOP can be directly accessed using this file path: "\\SMBGPA\addnmed5$\CAM SOPs\X. SOP Word Documents". This SOP explicates the steps clinical research coordinators will take to report identified distress to the study's trained clinical team who may conduct a follow-up safety assessment if indicated. Participants will be terminated from the protocol and referred for clinical care if the research team determines that their continued participation is unsafe or not in their best interest, (e.g., in circumstances where there is deterioration in their mental or physical health judged to require a change in medication or hospitalization). For any participant for whom we have concerns about emotional wellbeing that do not rise to the severity of an imminent safety risk, we will ask them if we can discuss this with their parents/guardians (if 16-17) and/or treater to help coordinate a plan for monitoring and treatment. If a participant <18 years of age is discontinued due to psychiatric concerns, we will refer them to clinical resources for follow-up care or consultation and we will ask them if this information can be disclosed to their parents/guardians to help coordinate and facilitate care. This procedure will also be used for ancillary participants who endorse imminent risk during their study visit.

To minimize exposure to study drug during pregnancy: There are no adequate and well-controlled studies of varenicline use in pregnant women. In animal studies, varenicline caused decreased fetal weight, increased auditory startle response, and decreased fertility in offspring. Varenicline should be used during pregnancy only if the potential benefit justifies the potential risk to the fetus. To be enrolled in this study, females of childbearing potential must be non-pregnant at enrollment and must agree to use effective contraception, such as an oral contraceptive agent, an intrauterine device, an implantable or injectable contraceptive, double barrier contraception or sexual abstinence for the duration of the study. The study drug will be discontinued immediately for any female subject who becomes pregnant during the treatment period of the study. These subjects will continue to attend study visits for follow-up.

To minimize abuse liability: Fewer than 1 out of 1,000 patients reported euphoria in clinical trials with varenicline. At higher doses (>2 mg/day), varenicline produced more frequent reports of gastrointestinal disturbances such as nausea and vomiting. There is no evidence of dose-escalation to maintain therapeutic effects in clinical studies, which suggests that tolerance does not develop. Abrupt discontinuation of varenicline was associated with an incr4ease in irritability and sleep disturbances in up to 3% of patients. This suggests that, in some patients, varenicline may produce mild physical dependence, which is not associated with addiction. In a human laboratory abuse liability study, a single oral dose of 1 mg varenicline did not produce any significant positive or negative subjective responses in smokers. In non-smokers, 1 mg varenicline produced an increase in some positive subjective effects, but this was accompanied by an increase in negative adverse effects, especially nausea. A single oral dose of 3 mg varenicline uniformly produced unpleasant subjective responses in both smokers and non-smokers.[38,77]

ii. <u>Nicotine Withdrawal Risks</u>: While it is well known that smoking (and vaping) cessation provides a large overall health benefit to patients, there are potential adverse, though transient, consequences of quitting smoking (and vaping). Many study participants who successfully abstain from nicotine vaping may experience nicotine withdrawal symptoms. Nicotine withdrawal symptoms include anxiety, restlessness, anger, irritability, sadness, difficulty concentrating, increase in appetite, weight gain, difficulty sleeping and craving for tobacco. Withdrawal symptoms begin within a few hours of abstinence, peak 24-48 hours after cessation, and may last up to 4 weeks. Nicotine withdrawal can cause slowing on EEG, decrease in cortisol and catecholamine levels, sleep EEG changes, and a decline in metabolic rate. In adults, the mean heart rate decline is about 8 beats per minute. As with all withdrawal syndromes, the severity varies among subjects. Withdrawal symptoms can also mimic or aggravate the symptoms of other psychiatric disorders or side effects of medications. Participants who are able to quit vaping nicotine are at risk for weight gain. Weight gain following nicotine cessation averages 3-5 kg.

<u>To minimize risk of nicotine withdrawal</u>: Half of participants will receive active varenicline, which will likely diminish withdrawal symptoms. All participants will receive written materials that list withdrawal symptoms that can be expected. Withdrawal symptoms are usually short-lived and never medically dangerous, and most abate after 1-2 weeks.

iii. <u>Psychosocial, Social and Legal Risks</u>: There are no significant psychological, social, or legal risks anticipated with participation in this study. There is a slight risk that the questionnaires and interviews may contribute to temporary discomfort when participants are asked about sensitive behaviors or personal feelings (e.g., drug use, vaping behavior, or mood), but, in our experience with similar studies, this risk is minimal. Participants will be reminded by study staff that they may omit any questions they do not wish to answer or discontinue their participation at any time with no penalty or repercussions. Research staff will provide referral information for treaters in the community to participants who request such information. As we will be meeting with participants regularly and administering validated mood and substance use questionnaires at each visit, we are able to monitor changes in endorsement of mental health symptoms. For any participant that experiences significant worsening of symptoms during study enrollment, we will share these results with them and will suggest that they seek professional support. For any participant for whom we have concerns about emotional wellbeing that do not rise to the severity of an imminent safety risk, we will ask them if we can discuss this with their parent (if under the age of 18) and/or treater to help coordinate a plan for monitoring and treatment.

<u>Psychosocial, Social, and Legal Protections</u>: We will maintain a Certificate of Confidentiality (CoC) from the National Institutes of Health for this study. The CoC protects the privacy of individuals who are subjects of research by establishing prohibitions on disclosure, in any Federal, State, or local civil, criminal, administrative, legislative, or other proceeding, of the names of research participants or any information, documents, or biospecimens that contain identifiable, sensitive information collected or used in research by an investigator or institution. We will use the CoC to resist any demands for information that would identify participants, except in the instances of child abuse and neglect, or harm to self or others.

Protecting the Anonymity of the Schools: Schools will not be identified and only minimal information about their characteristics will be released. When the results are published or discussed in conferences, no information will be included that would reveal an individual participant's identity or the name of the school at which the research was conducted. All data will be analyzed on a group level and not by individual participants.

iv. Confidentiality and Privacy Risks: Protecting the confidentiality and integrity of our research participants is a top priority for this and all MGH-based research projects. Data will be collected using REDCap (Research Electronic Data Capture)[78] tools hosted by MGH and Partners HealthCare. REDCap,REDCap Survey systems, and emocha provide secure, HIPAA compliant, web-based applications. Confidentiality will be maintained by numerically coding all data and by keeping all data in password protected databases. All study staff will be trained in protection of privacy of research participants and will be CITI certified. In addition, this study will maintain a Certificate of Confidentiality from the National Institutes of Health.

Confidentiality Protections: MGH's careful consent and data confidentiality procedures greatly minimize risk to privacy. Only MGH project investigators and authorized study staff will have access to data. Schools will not be provided any information regarding study participants, including cannabis use habits. Once collected, an individual's data will not be released to anyone outside of MGH authorized project staff. Confidentiality is further assured by assigning a unique identifier to each participant. There will be a restricted-access master list of names and other identifying information linked to the identification number. The master list will be kept in a locked cabinet and a password-protected computer file, separate from other data, along with other materials that have the participant's name (e.g., consent forms). No identifying information is listed on questionnaires or any other materials with data on them (e.g., drug tests). Self-report questionnaires will be administered via REDCap on Partners encrypted tablets, minimizing the risk of confidentiality breaches. Only authorized MGH project members will be allowed access to these tablets. Any data files in electronic format will be housed in our network server at the Center for Addiction Medicine at MGH and will be password protected so that only authorized project personnel have access to them. These files will not have participant names or identifying information attached to them. We will follow IRB standard procedures for protection of participant confidentiality in research studies that includes sending text messages that are relevant to the research study.

Limits of Confidentiality on Clinical Information (Emergency Protocol): While we are committed to maintaining confidentiality to the extent to which we are able, confidentiality is limited when there is a deemed imminent risk to oneself or others or reports of child and elder abuse. Study staff will inform participants during the consent process that in emergency situations (in which an individual is at immediate risk for harm) we will release information about the participant. Specifically, if a participant tells any member of study staff that they have intent and/or a plan to cause harm to self or others, study staff will start the Center Emergency protocol that includes a psychiatric evaluation by a licensed mental health professional and even calling 911. If the participant says that they have recurrent thoughts about harming themselves or someone else but does not have intent or plan to do so, study staff will ask participant permission to notify appropriate medical or counseling personnel, including the guidance counselor or therapist. If study staff learns about mood concerns or

problematic substance use, study staff will provide the participant with referral resources for follow-up consultation and care. The PI has prepared a comprehensive list of local and national resources for this purpose.

For participants under age 18: Parents and staff members at participating schools, will not be provided any information regarding study participants, including substance use habits, unless there is imminent risk of harm to self or others in the opinion of the PIs. In the consent/assent forms, both parents and participants are informed that only in emergency situations (in which an individual is at imminent risk of harm) would we release any information. Specifically, if a participant tells us that he/she has intent and/or a plan to cause harm self or other, we will tell the parents, and call 911. If the participant says that he/she has recurrent thoughts about harming him/herself or someone else but does not have intent or a plan to do so, we may notify parents and other appropriate medical or counseling personnel if clinically indicated in the opinion of the PIs. Finally, if a participant discusses details about ongoing child abuse, we will report such abuse to the Department of Child and Families directly. If we learn about mood concerns or problem substance use, we will provide the participant with local and trusted resources for follow-up consultation and we will ask him/her if this information can be disclosed to his/her parents to help coordinate and facilitate care.

Confidentiality of Drug Tests: The results of the qualitative and quantitative drug testing will be confidential. The only individuals who will have knowledge of the results of these tests are research staff directly working on the project. Information will be stored in a secure computer database that uses participant codes (rather than names) as identifiers.

v. Randomization in Clinical Trials Risk: Participants will be assigned to an intervention by chance, using computer generated random allocation code produced by the MGH Research Pharmacy. The study arm to which a participant is assigned may prove to be less effective than an alternate study arm.

VIII. POTENTIAL BENEFITS

a. Potential benefits to participating individuals

The study will potentially help individuals to stop vaping or substantially reduce their exposure to vaporized nicotine.

b. Potential benefits to society

The goal of this project is to test whether varenicline, the most effective treatment known for addiction to smoked tobacco, helps adolescent non-smokers who have developed nicotine dependence to quit vaping nicotine. This may benefit other adolescent nicotine vapers in the future and increase abstinence rates in this population by informing clinicians and adolescents who vape and their families of effective treatment options for nicotine vaping cessation. Given the prevalence of vaped nicotine use among adolescents who never smoked is rising more rapidly than any prior known drug, putting them at elevated risk for nicotine dependence, tobacco smoking, other drug use, and direct negative health effects of vaping, we believe that the few potential risks to participants in this research are outweighed by the potential benefits

IX. MONITORING AND QUALITY ASSURANCE

a. <u>Independent monitoring of source data</u>

All data management will be conducted in the offices of the PI at the MGH Center for Addiction Medicine (CAM) in Boston. Standard REDCap data collection forms for all proposed clinical rating scales will be used. Tablets with HIPAA compliant REDCap capability will be used to capture raw data from clinical rating scales entered by participants and study staff and self-reported substance use behavior. A study database will be designed by the PI and the data manager and maintained by the PI, the data manager and the research coordinators. The data manager will review the data weekly. Access to the database is restricted by password. The database will be protected by nightly backup on MGH servers. All data will be stored safely for at least 5 years after study completion.

b. <u>Safety monitoring</u>

An independent Data and Safety Monitoring Board (DSMB) will be appointed for this study, to assess the safety of the study by determining whether there is an unacceptable level of risk due to study medication, study procedures, and whether an increased number of adverse events occur. The DSMB will be established to analyze interim results to assess the safety of the study drug at regular intervals for the duration of the study by determining whether an increased number of adverse events occur among study participants receiving drug compared to participants receiving placebo.

The board will include a statistician, a smoking cessation expert, a pediatrician, and a psychiatrist. Each member of the DSMB will not otherwise be associated with the trial. Safety data will be reviewed by the Data and Safety Monitoring Board Data every 6 months after the recruitment period begins. The DSMB will receive summary reports on recruitment, retention and description of all adverse events and review them at each biannual DSMB meeting. The DSMB will receive all communication with the IRB and with the FDA. Subject information provided to the board will be identified only with study IDs to protect the confidentiality of subjects. The DSMB will assess interim results to determine whether the active drug treatment is associated with substantial risk, including higher rate of adverse outcomes when compared with the placebo group.

c. <u>Outcomes monitoring</u>

A DSMB Report written by the chair and approved by all members will be issued to the IRB, FDA, and the NIDA Project Officer after every DSMB meeting. The report will include, but may not be limited to, a synopsis of the trials, their progress to date, characteristics of participants enrolled, retention and disposition of study participants, quality assurance issues, regulatory issues, and reports of AEs and SAEs.

<u>Criteria for trial stopping rules</u>: If serious adverse events occur more frequently in the active group compared to the placebo group, at a 2-tailed p value of <0.05, then the DSMB will discuss with the PI and co-investigators whether the trial should be stopped

d. <u>Adverse event reporting guidelines</u>

Study staff, including co-investigators, research coordinators, and data managers, will meet weekly with the PIs and the Project Director during a weekly project management meeting to review study progress, including any adverse events.

All adverse events volunteered, observed, or solicited will be recorded in the AE CRF from the time the subject signs the informed consent up to and including Week 24. The PI will meet weekly with all study investigators to review the details of data acquisition and analysis as well as any minor problems. AEs will be assessed for each subject at every visit. All adverse events will be recorded and will include the dates of occurrence; severity; assessment of relationship to study drug; countermeasure(s); specific drug therapy used in countermeasure; and outcome. Adverse events will be reviewed by the PI who will complete an adverse event report form and submit this to the IRB within the required time frame in accordance with the IRB guidelines.

Reporting Adverse Events: The PI will report all adverse events experienced by the study subjects in accordance with HRC (Human Research Committee) guidelines to the Institutional Review board. Adverse events will also be reported by the principal investigator to the funding agency and to the FDA in accordance with IND regulations. The reporting of adverse events is the responsibility of the PIs, Drs. Evins and Schuster.

In case of serious adverse events (SAE's), the PI will report them within 24-hours by telephone, fax or email according to HRC guidelines, followed by a written report within 5 business days. SAEs that occur within 30 days after a subject's last dose of study medication that are categorized as definitely, probably or possibly relate[1]d to the study will be reported to the HRC and FDA. An annual report will be submitted to the HRC and the FDA of the progress of the trial. This will include individual study information and information on safety reports from the previous year.

X. REFERENCES

1. Johnston, L. D. *et al. Monitoring the Future National Survey Results on Drug Use, 1975-2018: Overview, Key Findings on Adolescent Drug Use*. (Institute for Social Research, 2019).

2. Wang, T. W. *et al.* Tobacco Product Use and Associated Factors Among Middle and High School Students — United States, 2019. *MMWR Surveill. Summ.* **68**, 1–22 (2019).

3. Barrington-Trimis, J. L. & Leventhal, A. M. Adolescents' Use of 'Pod Mod' E-Cigarettes - Urgent Concerns. *The New England journal of medicine* **379**, 1099–1102 (2018).

4. Kong, G. & Krishnan-Sarin, S. A call to end the epidemic of adolescent E-cigarette use. *Drug and Alcohol Dependence* **174**, 215–221 (2017).

5. Goriounova, N. A. & Mansvelder, H. D. Short- and Long-Term Consequences of Nicotine Exposure during Adolescence for Prefrontal Cortex Neuronal Network Function. *Cold Spring Harbor Perspectives in Medicine* **2**, a012120–a012120 (2012).
6. Smith, R. F., McDonald, C. G., Bergstrom, H. C., Ehlinger, D. G. & Brielmaier, J. M. Adolescent nicotine induces persisting changes in development of neural connectivity. *Neuroscience & Biobehavioral Reviews* **55**, 432–443 (2015).
7. Levine, A. *et al.* Molecular Mechanism for a Gateway Drug: Epigenetic Changes Initiated by Nicotine Prime Gene Expression by Cocaine. *Science Translational Medicine* **3**, 107ra109-107ra109 (2011).
8. Volkow, N. D. Epigenetics of Nicotine: Another Nail in the Coughing. *Science Translational Medicine* **3**, 107ps43-107ps43 (2011).
9. Young-Wolff, K. C. *et al.* Documentation of e-cigarette use and associations with smoking from 2012 to 2015 in an integrated healthcare delivery system. *Preventive Medicine* **109**, 113–118 (2018).
10. Curran, K. A., Burk, T., Pitt, P. D. & Middleman, A. B. Trends and Substance Use Associations With E-Cigarette Use in US Adolescents. *Clin Pediatr (Phila)* **57**, 1191–1198 (2018).
11. Soneji, S. *et al.* Association Between Initial Use of e-Cigarettes and Subsequent Cigarette Smoking Among Adolescents and Young Adults: A Systematic Review and Meta-analysis. *JAMA Pediatr* **171**, 788 (2017).
12. Printz, C. Researchers call teen vaping “one-way bridge” to smoking. *Cancer* **123**, 2188–2188 (2017).
13. Kandel, E. R. & Kandel, D. B. A Molecular Basis for Nicotine as a Gateway Drug. *N Engl J Med* **371**, 932–943 (2014).
14. Schramm-Sapyta, N. L., Walker, Q. D., Caster, J. M., Levin, E. D. & Kuhn, C. M. Are adolescents more vulnerable to drug addiction than adults? Evidence from animal models. *Psychopharmacology* **206**, 1–21 (2009).

15. Trivers, K. F., Phillips, E., Gentzke, A. S., Tynan, M. A. & Neff, L. J. Prevalence of Cannabis Use in Electronic Cigarettes Among US Youth. *JAMA Pediatr* **172**, 1097 (2018).

16. Rubinstein, M. L., Delucchi, K., Benowitz, N. L. & Ramo, D. E. Adolescent Exposure to Toxic Volatile Organic Chemicals From E-Cigarettes. *Pediatrics* **141**, e20173557 (2018).

17. Li, D. *et al.* Association of smoking and electronic cigarette use with wheezing and related respiratory symptoms in adults: cross-sectional results from the Population Assessment of Tobacco and Health (PATH) study, wave 2. *Tob Control* tobaccocontrol-2018-054694 (2019) doi:10.1136/tobaccocontrol-2018-054694.

18. Chaumont, M. *et al.* Short halt in vaping modifies cardiorespiratory parameters and urine metabolome: a randomized trial. *American Journal of Physiology-Lung Cellular and Molecular Physiology* **318**, L331–L344 (2020).

19. Chand, H. S., Muthumalage, T., Maziak, W. & Rahman, I. Pulmonary Toxicity and the Pathophysiology of Electronic Cigarette, or Vaping Product, Use Associated Lung Injury. *Front Pharmacol* **10**, 1619 (2019).

20. Krishnasamy, V. P. Update: Characteristics of a Nationwide Outbreak of E-cigarette, or Vaping, Product Use–Associated Lung Injury — United States, August 2019–January 2020. *MMWR Morb Mortal Wkly Rep* **69**, (2020).

21. Gotts, J. E., Jordt, S.-E., McConnell, R. & Tarran, R. What are the respiratory effects of e-cigarettes? *BMJ* l5275 (2019) doi:10.1136/bmj.l5275.

22. Bradford, L. E. *et al.* Danger in the vapor? ECMO for adolescents with status asthmaticus after vaping. *Journal of Asthma* **57**, 1168–1172 (2020).

23. Chadi, N., Hadland, S. E. & Harris, S. K. Understanding the implications of the "vaping epidemic" among adolescents and young adults: A call for action. *Substance Abuse* **40**, 7–10 (2019).

24. National Center for Chronic Disease, P., Health Promotion Office on, S., & Health. *Preventing Tobacco Use Among Youth and Young Adults: A Report of the Surgeon General*. (Centers for Disease Control and Prevention (US), 2012).
25. Kann, L. Youth Risk Behavior Surveillance — United States, 2015. *MMWR Surveill Summ* **65**, (2016).
26. Office of the Surgeon, G. *Smoking Cessation: A Report of the Surgeon General*. 70–72 https://www.hhs.gov/sites/default/files/2020-cessation-sgr-full-report.pdf (2020).
27. Etter, J. Are long-term vapers interested in vaping cessation support? *Addiction* **114**, 1473–1477 (2019).
28. Nearly half of young vapers resolve to quit e-cigarettes in 2020. https://truthinitiative.org/research-resources/quitting-smoking-vaping/nearly-half-young-vapers-resolve-quit-e-cigarettes-2020.
29. Barkat, S. S., Tellier, S. M. & Eloma, A. S. Varenicline for cessation from nicotine-containing electronic cigarettes. *Am J Health Syst Pharm* **76**, 1894–1895 (2019).
30. Sahr, M., Kelsh, S. E. & Blower, N. Pharmacist assisted vape taper and behavioral support for cessation of electronic nicotine delivery system use. *Clinical Case Reports* **8**, 100–103 (2020).
31. Quit vaping program sees high enrollment and engagement. https://truthinitiative.org/research-resources/quitting-smoking-vaping/quit-vaping-program-sees-high-enrollment-and-engagement.
32. Quit Vaping | Smokefree Teen. https://teen.smokefree.gov/quit-vaping.
33. Harvey, J. & Chadi, N. Preventing smoking in children and adolescents: Recommendations for practice and policy. *Paediatr Child Health* **21**, 209–214 (2016).
34. O'Dell, L. E. *et al.* Diminished nicotine withdrawal in adolescent rats: implications for vulnerability to addiction. *Psychopharmacology (Berl.)* **186**, 612–619 (2006).
35. Cahill, K., Stevens, S. & Lancaster, T. Pharmacological treatments for smoking cessation. *JAMA* **311**, 193–194 (2014).

36. Anthenelli, R. M. *et al.* Neuropsychiatric safety and efficacy of varenicline, bupropion, and nicotine patch in smokers with and without psychiatric disorders (EAGLES): a double-blind, randomised, placebo-controlled clinical trial. *The Lancet* **387**, 2507–2520 (2016).
37. Evins, A. E. *et al.* Neuropsychiatric Safety and Efficacy of Varenicline, Bupropion, and Nicotine Patch in Smokers With Psychotic, Anxiety, and Mood Disorders in the EAGLES Trial. *J Clin Psychopharmacol* **39**, 108–116 (2019).
38. Food & Drug, A. *Chantix (Varenicline tartrate)*. 2–52 https://www.fda.gov/media/124220/download (2018).
39. Faessel, H., Ravva, P. & Williams, K. Pharmacokinetics, safety, and tolerability of varenicline in healthy adolescent smokers: a multicenter, randomized, double-blind, placebo-controlled, parallel-group study. *Clin Ther* **31**, 177–189 (2009).
40. Gray, K. M. *et al.* Efficacy and Safety of Varenicline for Adolescent Smoking Cessation: A Randomized Clinical Trial. *JAMA Pediatr* **173**, 1146–1153 (2019).
41. Schuster R, E. E. Adolescent JUUL in Massachusetts' Schools, in Society for Research on Nicotine and Tobacco 2020 Annual Meeting. (2020).
42. McCabe, S. E., West, B. T. & McCabe, V. V. Associations Between Early Onset of E-cigarette Use and Cigarette Smoking and Other Substance Use Among US Adolescents: A National Study. *Nicotine Tob. Res.* **20**, 923–930 (2018).
43. Pourchez, J. & Forest, V. E-cigarettes: from nicotine to cannabinoids, the French situation. *The Lancet Respiratory Medicine* **6**, e16 (2018).
44. Muramoto, M. L., Leischow, S. J., Sherrill, D., Matthews, E. & Strayer, L. J. Randomized, Double-blind, Placebo-Controlled Trial of 2 Dosages of Sustained-Release Bupropion for Adolescent Smoking Cessation. *Arch Pediatr Adolesc Med* **161**, 1068 (2007).

45. Rollema, H. *et al.* Pharmacological profile of the alpha4beta2 nicotinic acetylcholine receptor partial agonist varenicline, an effective smoking cessation aid. *Neuropharmacology* **52**, 985–994 (2007).
46. Chantix (Varenicline): Uses, Dosage, Side Effects, Interactions, Warning. RxList.
47. Quit Smoking. https://www.lung.org/quit-smoking.
48. Fanshawe, T. R. *et al.* Tobacco cessation interventions for young people. *Cochrane Database Syst Rev* **11**, CD003289 (2017).
49. Fiore MC, J. C., Baker TB, et al. *Treating Tobacco Use and Dependence: 2008 Update. Clinical Practice Guideline.* (U.S. Department of Health and Human Services. Public Health Service, 2008).
50. Clinic, M. Mayo Clinic 5-E Model of Wellness Coaching 2019. https://wellnesscoachtraining.mayo.edu/why-us/.
51. Quit vaping program sees high enrollment and engagement. *Truth Initiative*.
52. Initiative, T. T. This is Quitting. *Truth Initiative*.
53. PhenX Toolkit: Domains.
54. Hyland, A. *et al.* Design and methods of the Population Assessment of Tobacco and Health (PATH) Study. *Tob Control* **26**, 371–378 (2017).
55. PhenX Toolkit: Protocols. https://www.phenxtoolkit.org/protocols/view/710501.
56. Posner, K. *et al.* The Columbia–Suicide Severity Rating Scale: Initial Validity and Internal Consistency Findings From Three Multisite Studies With Adolescents and Adults. *AJP* **168**, 1266–1277 (2011).
57. Protocol - Flavor Preference - e-cigarettes - Youth.
58. Schneller, L. M. *et al.* Use of Flavored E-Cigarettes and the Type of E-Cigarette Devices Used among Adults and Youth in the US-Results from Wave 3 of the Population Assessment of Tobacco and Health Study (2015-2016). *Int J Environ Res Public Health* **16**, (2019).
59. PhenX: Host: Social/Cognitive measures for tobacco regulatory research | Tobacco Control.

60. Piper, M. E., Baker, T. B., Benowitz, N. L., Smith, S. S. & Jorenby, D. E. E-cigarette Dependence Measures in Dual Users: Reliability and Relations with Dependence Criteria and E-Cigarette Cessation. *Nicotine Tob. Res.* (2019) doi:10.1093/ntr/ntz040.
61. Saunders, J. B., Aasland, O. G., Babor, T. F., De La Fuente, J. R. & Grant, M. Development of the Alcohol Use Disorders Identification Test (AUDIT): WHO Collaborative Project on Early Detection of Persons with Harmful Alcohol Consumption-II. *Addiction* **88**, 791–804 (1993).
62. Noorbakhsh, S. *et al.* Psychometric properties of the Alcohol Use Disorders Identification Test (AUDIT) and prevalence of alcohol use among Iranian psychiatric outpatients. *Substance Abuse Treatment, Prevention, and Policy* **13**, 5 (2018).
63. Adamson, S. J. & Sellman, J. D. A prototype screening instrument for cannabis use disorder: the Cannabis Use Disorders Identification Test (CUDIT) in an alcohol-dependent clinical sample. *Drug and Alcohol Review* **22**, 309–315 (2003).
64. Dowd, A. N., Motschman, C. A. & Tiffany, S. T. Development and Validation of the Questionnaire of Vaping Craving. *Nicotine Tob. Res.* **21**, 63–70 (2019).
65. Cappelleri, J. C. *et al.* Revealing the multidimensional framework of the Minnesota nicotine withdrawal scale. *Curr Med Res Opin* **21**, 749–760 (2005).
66. Trivedi, M. H. *et al.* Concise Health Risk Tracking Scale: A Brief Self-Report and Clinician Rating of Suicidal Risk. *J. Clin. Psychiatry* **72**, 757–764 (2011).
67. Mayes, T. L. *et al.* Psychometric properties of the concise health risk tracking (CHRT) in adolescents with suicidality. *Journal of Affective Disorders* **235**, 45–51 (2018).
68. Lin, A. *et al.* Validation of a short adaptation of the Mood and Anxiety Symptoms Questionnaire (MASQ) in adolescents and young adults. *Psychiatry Res* **215**, 778–783 (2014).
69. Shafer, A. B. & Temple, J. M. Factor Structure of the Mental Health Statistics Improvement Program (MHSIP) Family and Youth Satisfaction Surveys. *J Behav Health Serv Res* **40**, 306–316 (2013).

70. Cyders, M. A., Littlefield, A. K., Coffey, S. & Karyadi, K. A. Examination of a short English version of the UPPS-P Impulsive Behavior Scale. *Addictive Behaviors* **39**, 1372–1376 (2014).

71. Wechsler, D. *Wechsler Test of Adult Reading*. (Pearson, 2001).

72. Gioia, G. A., Guy, S. C., Isquith, P. K. & Kenworthy, L. *Behavior rating inventory of executive function*. (Psychological Assessment Resources, 1996).

73. Buuren, S. van & Groothuis-Oudshoorn, K. mice: Multivariate Imputation by Chained Equations in R. *J. Stat. Soft.* **45**, (2011).

74. Foulds, J. *et al.* Development of a questionnaire for assessing dependence on electronic cigarettes among a large sample of ex-smoking E-cigarette users. *Nicotine Tob. Res.* **17**, 186–192 (2015).

75. R Core Team. R: A language an d environment for statistical computing (version 4.1.1).

76. Halekoh, U., Højsgaard, S. & Yan, J. The R Package geepack for Generalized Estimating Equations. *J. Stat. Soft.* **15**, (2006).

77. Faessel, H. M. *et al.* Multiple-dose pharmacokinetics of the selective nicotinic receptor partial agonist, varenicline, in healthy smokers. *J Clin Pharmacol* **46**, 1439–48 (2006).

78. Harris, P. A. *et al.* Research electronic data capture (REDCap)—A metadata-driven methodology and workflow process for providing translational research informatics support. *Journal of Biomedical Informatics* **42**, 377–381 (2009).

79. Hunt & Allen (2023). Friendship Instability in Adolescence: Links to Attachment, Romantic Relationship Quality, and Physical Health in Adulthood. [Unpublished Thesis], University of Virginia.

80. McCarty, C., Lubbers, M. J., Vacca, R., & Molina, J. L. (2019). *Conducting personal network research: A practical guide*. Guilford Publications.

81. Poulin & Chan (2010). Friendship stability and change in childhood and adolescence. *Developmental Review, 30,* 257-272. Alessandra

82. Ryan, L. (2021). Telling network stories: researching migrants' changing social relations in places over time. *Global Networks*, *21*(3), 567-584.
