# Supplementary material for: Cannabis Use and Nicotine Vaping Cessation Outcomes: A Secondary Analysis of a Randomized Clinical Trial
Source: JAMA Netw Open. 2025 Dec 12;8(12):e2547799. doi: 10.1001/jamanetworkopen.2025.47799 (PMC12701513; doi:10.1001/jamanetworkopen.2025.47799)
Supplement: Supplement 2. — eTable 1. Sensitivity Analysis Results for Association Between Cannabis Use and Abstinence Excluding Varenicline Group eTable 2. Sensitivity Analysis Results for Association Between Cannabis Use and Abstinence Using Continuous Week 9-12 Abstinence eTable 3. Sensitivity Analysis of Interaction Models of Varenicline by Baseline Cannabis Use, Using Continuous Week 9-12 Abstinence [file jamanetwopen-e2547799-s002.pdf]

Supplemental Online Content

Gilman JM, Cather C, Reeder HT, et al. Association between cannabis use and nicotine vaping cessation outcomes. *JAMA Netw Open.* 2025;8(12):e2547799. doi:10.1001/jamanetworkopen.2025.47799

eTable 1. Sensitivity Analysis Results for Association Between Cannabis Use and Abstinence Excluding Varenicline Group

eTable 2. Sensitivity Analysis Results for Association Between Cannabis Use and Abstinence Using Continuous Week 9-12 Abstinence

eTable 3. Sensitivity Analysis of Interaction Models of Varenicline by Baseline Cannabis Use, Using Continuous Week 9-12 Abstinence

This supplemental material has been provided by the authors to give readers additional information about their work.

© 2025 Gilman JM et al. *JAMA Network Open*.

eTable 1. Sensitivity analysis results for association between cannabis use and abstinence excluding Varenicline group.

**Multivariable model:[a] Use days per week**	**Adjusted OR (95% CI)**	**p-value[b]**
Cannabis use days per week		0.88
0	Reference	
>0 and <4	1.16 (0.44-3.13)	
4+	0.91 (0.32-2.58)	
AUDIT (5-unit increase)	0.91 (0.56-1.41)	0.67
Multivariable model:[a] Categorical CUDIT	Adjusted OR (95% CI)	p-value[b]
CUDIT (categorical)		0.55
0-7	Reference	
8-11	0.56 (0.15-1.76)	
12+	1.09 (0.46-2.55)	
AUDIT (5-unit increase)	0.94 (0.6-1.44)	0.78
Multivariable model:[a] Continuous CUDIT	Adjusted OR (95% CI)	p-value[b]
CUDIT (5-unit increase)	0.98 (0.73-1.28)	0.88
AUDIT (5-unit increase)	0.90 (0.57-1.38)	0.65

[a]Each multivariable model also adjusts for sex and age.

[b]p-values for categorical cannabis use variables from overall likelihood ratio tests, and p-values for AUDIT and continuous CUDIT variables from Wald tests.

© 2025 Gilman JM et al. *JAMA Network Open*.

eTable 2. Sensitivity analysis results for association between cannabis use and abstinence using continuous week 9-12 abstinence.

**Multivariable model:[a] Use days per week**	**Adjusted OR (95% CI)**	**p-value[b]**
Cannabis use days per week		0.07
0	Reference	
>0 and <4	2.52 (1.10-6.03)	
4+	1.27 (0.49-3.29)	
AUDIT (5-unit increase)	0.98 (0.64-1.48)	0.93
Varenicline (vs. Placebo/EUC)	12.63 (6.38-26.37)	<0.001
Multivariable model:[a] Categorical CUDIT	Adjusted OR (95% CI)	p-value[b]
CUDIT (categorical)		0.76
0-7 (low risk)	Reference	
8-11 (moderate risk)	1.00 (0.38-2.55)	
12+ (high risk)	1.32 (0.60-2.93)	
AUDIT (5-unit increase)	1.02 (0.67-1.52)	0.93
Varenicline (vs. Placebo/EUC)	13.66 (6.99-28.21)	<0.001
Multivariable model:[a] Continuous CUDIT	Adjusted OR (95% CI)	p-value[b]
CUDIT (5-unit increase)	1.11 (0.86-1.42)	0.43
AUDIT (5-unit increase)	1.00 (0.66-1.49)	1.0
Varenicline (vs. Placebo/EUC)	13.64 (6.98-28.12)	<0.001

[a]Each multivariable model also adjusts for sex and age.

[b]p-values for categorical cannabis use variables from overall likelihood ratio tests, and p-values for AUDIT, Varenicline, and continuous CUDIT variables from Wald tests.

© 2025 Gilman JM et al. *JAMA Network Open*.

Table S3: Sensitivity analysis of interaction models of varenicline by baseline cannabis use, using continuous week 9-12 abstinence

	Group-specific Adjusted OR (95% CI)			
Multivariable model:[a] Use days per week	0	>0 and <4	4+	Interaction p-value[b]
Varenicline (vs. Placebo/EUC)	10.63 (2.86-52.22)	13.49 (5.09-39.50)	13.07 (3.62-56.14)	0.96
Multivariable model:[a] Categorical CUDIT	0-7	8-11	12+	
Varenicline (vs. Placebo/EUC)	11.97 (4.78-33.62)	48.67 (7.31-990.85)	10.03 (3.21-34.35)	0.39

[a]Each multivariable model also adjusts for sex, age, and continuous AUDIT score, and includes varenicline-cannabis interaction terms used to calculate group-specific adjusted ORs.

[b]Interaction p-values for categorical cannabis use variables calculated from an overall likelihood ratio test of the interaction terms.

© 2025 Gilman JM et al. *JAMA Network Open*.
