# Supplementary material for: Cannabis Use and Nicotine Vaping Cessation Outcomes: A Secondary Analysis of a Randomized Clinical Trial
Source: JAMA Netw Open. 2025 Dec 12;8(12):e2547799. doi: 10.1001/jamanetworkopen.2025.47799 (PMC12701513; doi:10.1001/jamanetworkopen.2025.47799)
Supplement: Supplement 3. — Data Sharing Statement [file jamanetwopen-e2547799-s003.pdf]

Data Sharing Statement

Gilman. Cannabis Use and Nicotine Vaping Cessation Outcomes. *JAMA Netw Open*.
Published December 12, 2025. doi:10.1001/jamanetworkopen.2025.47799

Data
Additional Information: ClinicalTrials.gov NCT05367492
https://clinicaltrials.gov/study/NCT05367492
Data available: Yes
Data types: Data dictionary, Other (please specify), Deidentified participant data
Additional Information: Analytic code
How to access data: The data that support the findings of this study are available from the corresponding author upon request. Requests should be sent to aeevins@mgh.harvard.edu. Data will include de-identified individual patient-level data, a data dictionary, and analytic code. Investigators proposing to use the data must execute a data use agreement with Massachusetts General Hospital and have approval from an Institutional Review Board (IRB), Independent Ethics Committee (IEC), or Research Ethics Board (REB), as applicable, before data is shared. Data will be available within three months of publication.
When available: With publication

Supporting Documents
Document types: None

Additional Information
Who can access the data: The data that support the findings of this study will be made available to individuals who request the data from the corresponding author.
Types of analyses: For individuals seeking further inquiry.
Mechanisms of data availability: With investigator support.
